# The Evolutionary Basis of Translational Accuracy in Plants

**DOI:** 10.1534/g3.117.040626

**Published:** 2017-05-22

**Authors:** Salvatore Camiolo, Gaurav Sablok, Andrea Porceddu

**Affiliations:** * Dipartimento di Agraria, Sezione di Agronomia, Coltivazione Erbacee e Genetica, Università degli Studi di Sassari, 07100, Italy; † Plant Functional Biology and Climate Change Cluster, University of Technology Sydney, New South Wales 2007, Australia

**Keywords:** translational accuracy, codon bias, protein domains, RNA folding

## Abstract

Mistranslation errors compromise fitness by wasting resources on nonfunctional proteins. In order to reduce the cost of mistranslations, natural selection chooses the most accurately translated codons at sites that are particularly important for protein structure and function. We investigated the determinants underlying selection for translational accuracy in several species of plants belonging to three clades: Brassicaceae, Fabidae, and Poaceae. Although signatures of translational selection were found in genes from a wide range of species, the underlying factors varied in nature and intensity. Indeed, the degree of synonymous codon bias at evolutionarily conserved sites varied among plant clades while remaining uniform within each clade. This is unlikely to solely reflect the diversity of tRNA pools because there is little correlation between synonymous codon bias and tRNA abundance, so other factors must affect codon choice and translational accuracy in plant genes. Accordingly, synonymous codon choice at a given site was affected not only by the selection pressure at that site, but also its participation in protein domains or mRNA secondary structures. Although these effects were detected in all the species we analyzed, their impact on translation accuracy was distinct in evolutionarily distant plant clades. The domain effect was found to enhance translational accuracy in dicot and monocot genes with a high GC content, but to oppose the selection of more accurate codons in monocot genes with a low GC content.

The translation of genes into proteins is a core activity shared by all living organisms. This molecular process requires the commitment of significant resources, including energy and raw materials (Stoebel *et al.* 2008). For this reason, many species have evolved mechanisms to optimize translational efficiency and accuracy. Translational efficiency can be defined as the capacity to produce large amounts of protein while minimizing the associated costs (Dekel and Alon 2005), whereas translational accuracy can be defined as increasing the probability that the resulting protein is error free (Drummond and Wilke 2008; Zhou *et al.* 2009). The redundancy of the genetic code means that most amino acids can be specified by more than one (synonymous) codon and this provides an evolutionary mechanism to refine the efficiency and accuracy of translation while maintaining the same amino acid sequence. Synonymous codons are not used with the same frequency in different genes and species—a phenomenon known as codon bias (Plotkin and Kudla 2011). Although this can in some cases reflect neutral genetic drift (Li 1987), selection pressure may also be responsible (Komar *et al.* 1999; Kudla *et al.* 2009). The tRNAs representing synonymous codons are not equally abundant in the cytosol and some may even be absent, so the enrichment of genes for codons matching the most abundant tRNAs (known as optimal codons) can increase the speed and efficiency of translation and protein accumulation (Wright 2004). The association between codon bias and gene expression level is generally considered a signature of selection for translational efficiency (dos Reis *et al.* 2004). Similarly, optimal codons are often concentrated in gene sequences that encode structurally or functionally critical residues, which tend to be conserved during evolution, and this is considered a signature of selection for translational accuracy (Drummond and Wilke 2008). The incorporation of a suboptimal tRNA featuring a one-base mismatch relative to the codon (a near-cognate tRNA) within such sites may attract severe energy-related costs due to the increased likelihood of synthesizing a nonfunctional protein or a misfolded protein that interacts unfavorably with other cellular components to promote aggregation (Gingold and Pilpel 2011). Selection for translational accuracy was first detected and quantified in *Drosophila melanogaster* (Akashi 1994) and was later confirmed in other species, such as *Escherichia coli* (Stoletzki and Eyre-Walker 2007) and several eukaryotes (Drummond and Wilke 2008).

Although translational selection has been widely studied in plants (Wang and Roossinck 2006), few of these studies have considered signatures of translational accuracy and the impact of additional factors (Porceddu *et al.* 2013). For example, the selection of mRNA secondary structures (whose organization relies on the formation of stems and loops) may potentially be involved in translational regulation (Stoletzki 2008). Indeed, stem sequences are expected to be GC-rich because G/C pairs are more stable than A/T pairs (Chan *et al.* 2009), but most of the optimal codons in plants end with GC, so the accumulation of optimal codons around conserved protein sites may reflect the formation of secondary structures that provide false signals of translational accuracy. Similarly, transcript portions corresponding to protein domains may be more GC-rich because they need to form complex structures that hinder the ribosome, allowing sufficient time for the nascent protein to fold correctly (Vandivier *et al.* 2014; Homma *et al.* 2016). Again, the enrichment of conserved sites within such regions may introduce false signatures of translational accuracy.

In order to better understand the determinants underlying selection for translational accuracy in plants, we extended our previous study (Porceddu *et al.* 2013) to several species belonging to three clades: Brassicaceae, Fabidae, and Poaceae. After investigating the conservation of translational accuracy during evolution, the underlying mechanism was investigated in detail by controlling for additional factors, such as selection for mRNA secondary structure and the potential GC enrichment of transcript portions coding for protein domains. The reported results clearly underline the influence of these factors on the precise detection of the translational accuracy signature in higher plants, resulting in effects that are evolutionary conserved within species belonging to the same clade. In this regard, our results suggest careful consideration of the general GC content increase within domains when investigating the occurrence of GC3-rich optimal codons in conserved portions of the transcripts.

## Materials and Methods

### Sequences and expression data

FASTA format genomic sequences together with the corresponding gff3 format genic annotation files were downloaded from the Phytozome website (https://phytozome.jgi.doe.gov/pz/portal.html). The species included in this study were comprised of five Brassicaceae, namely *Arabidopsis thaliana* (AT; v. 167, TAIR9), *Arabidopsis lyrata* (AL; v. 107), *Brassica rapa* (BR; v. 277), *Capsella rubella* (CR; v. 183) and *Eutrema salsugineum* (ES; v. 173); five Fabidae, namely *Fragaria vesca* (FV; v. 226), *Glycine max* (GM; v. 275), *Medicago truncatula* (MT; v. 285), *Prunus persica* (PP; v. 139), and *Phaseolus vulgaris* (PV; v. 218); and four Poaceae, namely *Brachypodium distachyon* (BD; v. 283), *Oryza sativa* (OS; v. 204), *Sorghum bicolor* (SB; v. 255), and *Zea mays* (ZM; v. 284). The two-letter abbreviations for these species are used when we describe interspecies comparisons.

Coding sequences (CDS), mature mRNA sequences, and protein sequences were generated by computing the annotation information using gff2sequence (Camiolo and Porceddu 2013). Only CDS beginning with a valid start codon (ATG), ending with a canonical stop codon (TGA, TAA, or TAG), and featuring full nucleotide triplets (*e.g.*, the sequence length was a multiple of three) were used for further analysis. For monocots, the sequence datasets were also assigned as high-GC genes (HGC; GC ≥ 60%) and low-GC genes (LGC; GC < 60%) (Mukhopadhyay *et al.* 2008).

Expression data were retrieved from PlexDB (Dash *et al.* 2012) and the NCBI GEO website for *A. thaliana*, *M. truncatula*, *O. sativa*, and *Z. mays* (Supplemental Material, Table S1). Only genes with expression levels >100 were considered to be expressed and the corresponding values were log-transformed to minimize the effect of outliers. The average expression level for each gene was calculated by considering only experiments in which that gene was actually expressed (Camiolo *et al.* 2009).

### Identification of orthologs and protein alignments

Orthologs were identified using OrthoMCL (Li *et al.* 2003) with an inflation value of four. Orthologs with an alignment coverage achieving >60% identity were retained for further analysis. Alignment files were created for each species by comparing the proteins with the orthologs in all the other species within the evolutionary clade, using MUSCLE v3.8.31 (Edgar 2004) with default parameters. For each pairwise comparison, we chose the orthologous pairs based on the BLAST *e*-value (Altschul *et al.* 1990). In cases of one to many or many to many relationships the pair of loci with the best *e*-values were analyzed. For three species comparisons (see *Phylogeny-based* *dataset*) we used only trios involving reciprocal best orthologous loci. Amino acids that did not vary in the alignment of orthologs were considered to be conserved.

### Identification and analysis of protein domains

Protein sequences were scanned against the PFAM-A database (Finn *et al.* 2016) using HMMscan (http://hmmer.org/). The nucleotide composition of domain and nondomain segments of transcripts were analyzed using domain coordinates to extract domain sequences together with their 5′ and 3′ flanking regions from each CDS. Only regions that were at least 100 amino acids long at this stage were retained for further analysis. Any 5′ and 3′ flanking regions featuring additional domain portions were removed from the final dataset. The GC (guanine + cytosine) and GC3 (guanine + cytosine at the third codon position) values were computed for the last 100 codons of the 5′ flanking domain region, the first 100 codons of the 3′ flanking domain, and the first and last 100 codons of each domain, using a sliding window approach (window step = 3 bases, window size = 9 bases). Values from the domain flanking regions were pooled and flagged as nondomain, whereas values from the first and last portion of domains were pooled together and flagged as domain. Wilcoxon tests were applied to determine whether GC and GC3 values diverged significantly between nondomain and domain regions.

### Analysis of mRNA folding

The mRNA sequences were processed using the Vienna RNAfold package (Lorenz *et al.* 2011) with default parameters, and each gene was annotated at the single nucleotide level as unpaired loop regions and weakly or strongly paired stem regions. Codons with a strongly paired third base in the predicted folding model were described as S-codons and all others were described as L-codons.

### tRNA gene copy number and identification of optimal codons

The tRNA gene copy number was determined using tRNAscan (Lowe and Eddy 1997) with default parameters. When the organelle genome was annotated, the corresponding scaffold was removed before running the analysis. If such information was not available, a BLASTn search was performed on the entire genome *vs.* the mitochondrial and plastid sequences of *A. thaliana* and the corresponding scaffolds were discarded. Because the tRNA gene copy number is associated with genome size, the relative tRNA-RSCU value was calculated by normalizing the number of tRNA copies in a manner analogous to calculating the relative synonymous codon usage (RSCU) (Sharp *et al.* 1986):

$${\text{RSCU}}_{i}^{\text{tRNA}}=\frac{\sum_{t}{\text{tRNA}}_{i}^{\mathrm{copies}}}{n\times \sum_{j}^{n}{\text{tRNA}}_{j}^{\mathrm{copies}}},$$

where *n* is the degeneracy of the corresponding codon family.

Optimal codons were identified for four plant species spanning the three investigated clades (*A. thaliana*, *M. truncatula*, *O. sativa*, and *Z. mays*) using Seforta (Camiolo *et al.* 2014). For each species, the 20% most expressed and least expressed genes were extracted. Codons that proved to be statistically more frequent in strongly expressed genes were considered optimal codons. Seforta yields an odds ratio value for each codon that is >1 when it is used preferentially in strongly expressed genes and ≤1 otherwise. The significance of the odds ratio was taken into account when calling the optimal codons. For the monocots species, the analysis was carried out separately for the HGC and LGC datasets.

### Analysis of codon enrichment and translational accuracy

Seforta was used to investigate the enrichment of each codon compared to its synonymous peers at the constrained and unconstrained sites. We defined constrained sites as those unchanged in protein coding alignments and unconstrained sites as those showing divergence in at least one species. Briefly, Seforta treats each codon as optimal while considering others within the same synonymous family as nonoptimal. The optimal and nonoptimal codons representing conserved and nonconserved sites in each gene are computed to generate a 2 × 2 matrix. The Mantel–Haenszel (MH) test was then used to pool the matrices and to calculate a cumulative odds ratio for each codon (Akashi 1994). Computation was performed so that codons were assigned an odds ratio >1 if they are preferentially used at conserved sites. We refer to this approach as the original Akashi test. Because this approach identifies unconstrained sites based on the presence of an amino acid substitution, it is possible that the codon bias at these sites is influenced by the nonsynonymous substitution rate. Two further datasets were therefore generated to control for this effect.

#### Phylogeny-based dataset:

**Figure 1:**
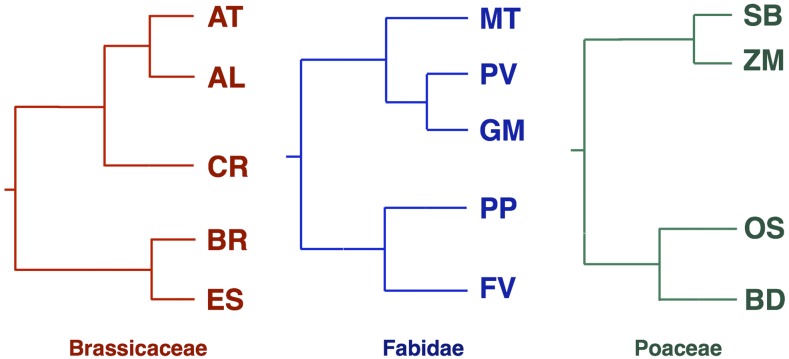
Phylogenetic trees corresponding to the clades we analyzed: *Arabidopsis thaliana* (AT), *Arabidopsis lyrata* (AL), *Brassica rapa* (BR), *Capsella rubella* (CR), *Eutrema salsugineum* (ES), *Fragaria vesca* (FV), *Glycine max* (GM), *Medicago truncatula* (MT), *Prunus persica* (PP), *Phaseolus vulgaris* (PV), *Brachypodium distachyon* (BD), *Oryza sativa* (OS), *Sorghum bicolor* (SB), and *Zea mays* (ZM).

We used an alignment of three species (*e.g.*, the principal species under analysis, a sister species, and an outgroup species within the same family) to infer the lineage in which the change had occurred. For example, in order to study the translational accuracy in AT (Figure 1), we produced alignment for the species AT (species under study), AL (sister species), and BR (outgroup within the family). We considered only sites that were inferred to have mutated in the sister species, thus ruling out the hypothesis that codon bias at these sites could be influenced by nonsynonymous changes in the principal species. We refer to this approach as the phylogeny-based Akashi test (see Table S2 for the species triplets used).

#### Optimality-tied dataset:

We used a pairwise alignment between orthologous loci, and considered only nonconserved codons that met the following criteria: (i) the same (third) silent base and (ii) the same favored base. For codon pairs that differed at two bases (first and second base), we checked that both the above criteria were met by all potential intermediate changes in all reconstructed pathways between the two codons. We refer to this approach as the optimality-tied Akashi test.

Seforta codon enrichment analysis has been adapted to study the differential use of synonymous codons in a variety of scenarios. In this investigation, we calculated the preferential use of each codon in domain *vs.* nondomain regions and in stem *vs.* loop regions. Similarly, we investigated the differential use of synonymous codons in conserved sites within domain *vs.* nondomain regions and stem *vs.* loop regions.

Seforta was also used to investigate signatures of translational accuracy in *A. thaliana*, *M. truncatula*, *O. sativa*, and *Z. mays*. As originally reported (Camiolo *et al.* 2014), Seforta implements the Akashi test to investigate whether optimal codons are used preferentially at conserved *vs.* nonconserved sites. Again, an odds ratio was generated, indicating selection for translational accuracy when the value is significant and >1.

### Statistical analysis

The effective number of codons was calculated using codonw (http://codonw.sourceforge.net/). The fraction of optimal codons (Fop) (Ikemura 1981) was computed using *in situ* python software. Heat maps and scatterplots, together with the reported statistical analysis (*e.g.*, Pearson correlation, Spearman correlation, and Wilcoxon and MH tests), were generated using R software (R Core Team, 2014).

### Positional effect analysis

Translational accuracy and the frequency of optimal codons were calculated along the transcript by using an adjacent windows approach. Briefly, each alignment was split into windows of five amino acids. Alignment portions for all genes within the same window were pooled in a superalignment. At the same time, a transcript supersequence was assembled for each window by retrieving the corresponding codons from the original CDS. The resulting artificial alignments and CDS were analyzed by Seforta using the Akashi test and by applying a custom script to calculate the Fop statistics.

### Data availability

File S1 contains a detailed description of all the supplemental files. File S2 and File S3 contain the ortholog genes used in this study. File S4 includes the coordinates of the protein domains for all the analyzed species. File S5 reports pseudoalignments resulting from the computation of stem and loop structures within the transcripts.

## Results

### Codon choice and evolutionary constraints at the encoded site

Codons that are accurately translated should be found preferentially at conserved sites because these are more likely to affect fitness in cases of mistranslation (Akashi 1994). A common method to highlight signatures of translational accuracy is based on the computation of codon usage at evolutionarily conserved and variable protein sites. However, a number of potential confounding effects can influence this type of analysis, including the gene expression level, mutational bias, and nonsynonymous mutations. Because codon usage is evaluated separately for each CDS, the overall effects of mutational bias or gene expression level can be ruled out *a priori* (Akashi 1994). However, a similar conclusion cannot be reached for the effect of nonsynonymous substitutions. Indeed, as pointed out by Lipman and Wilbur (1984), the codon bias at diverged sites may depend on the codon usage before the mutation occurred rather than synonymous codon selection. Such an effect should be particularly relevant for “young” changes for which mutation/selection had, eventually, limited opportunities to reestablish the appropriate favored/unfavored base. We therefore attempted to control for such effects by discriminating among unconstrained sites on the basis of the lineage in which the mutation occurred. In practice, the changes that occurred in this principal lineage were excluded and only those sites that were inferred to have mutated along the sister lineage were considered (phylogeny-based Akashi test, see *Materials and Methods*). The codon choice at these sites in the investigated species is expected to be influenced solely by natural selection and not by nonsynonymous substitution bias.

We identified conserved and variable amino acid residues in orthologs from several evolutionary clades (Figure 1) among the Brassicaceae (representing the malvids: *A. thaliana*, *A. lyrata*, *B. rapa*, *C. rubella*, and *E. salsugineum*), Fabidae (representing the fabids: *F. vesca*, *G. max*, *M. truncatula*, *P. persica*, and *P. vulgaris*) and Poaceae (representing the monocots: *B. distachyon*, *O. sativa*, *S. bicolor*, and *Z. mays*). Codon enrichment analysis at the conserved sites was carried out by computing an odds ratio in accordance with the MH test.

For each species, we calculated a mean odds ratio for each codon by averaging its values in all the comparisons we analyzed. For example, the odds ratio for codon TTC was calculated in *A. thaliana* by averaging the odds ratio values that were found for this codon when analyzing the conserved sites in the alignments [(AT-AL)-BR], [(AT-CR)-BR], [(AT-ES)-BR], and [(AT-BR)-ES], and considering as unconstrained sites those inferred to have changed in the sister species. This approach allowed us to estimate the variability of the calculated odds ratio with respect to the species used for each computation. Average values >1 indicated the overrepresentation of the codon at conserved sites compared to variable sites.

**Figure 2:**
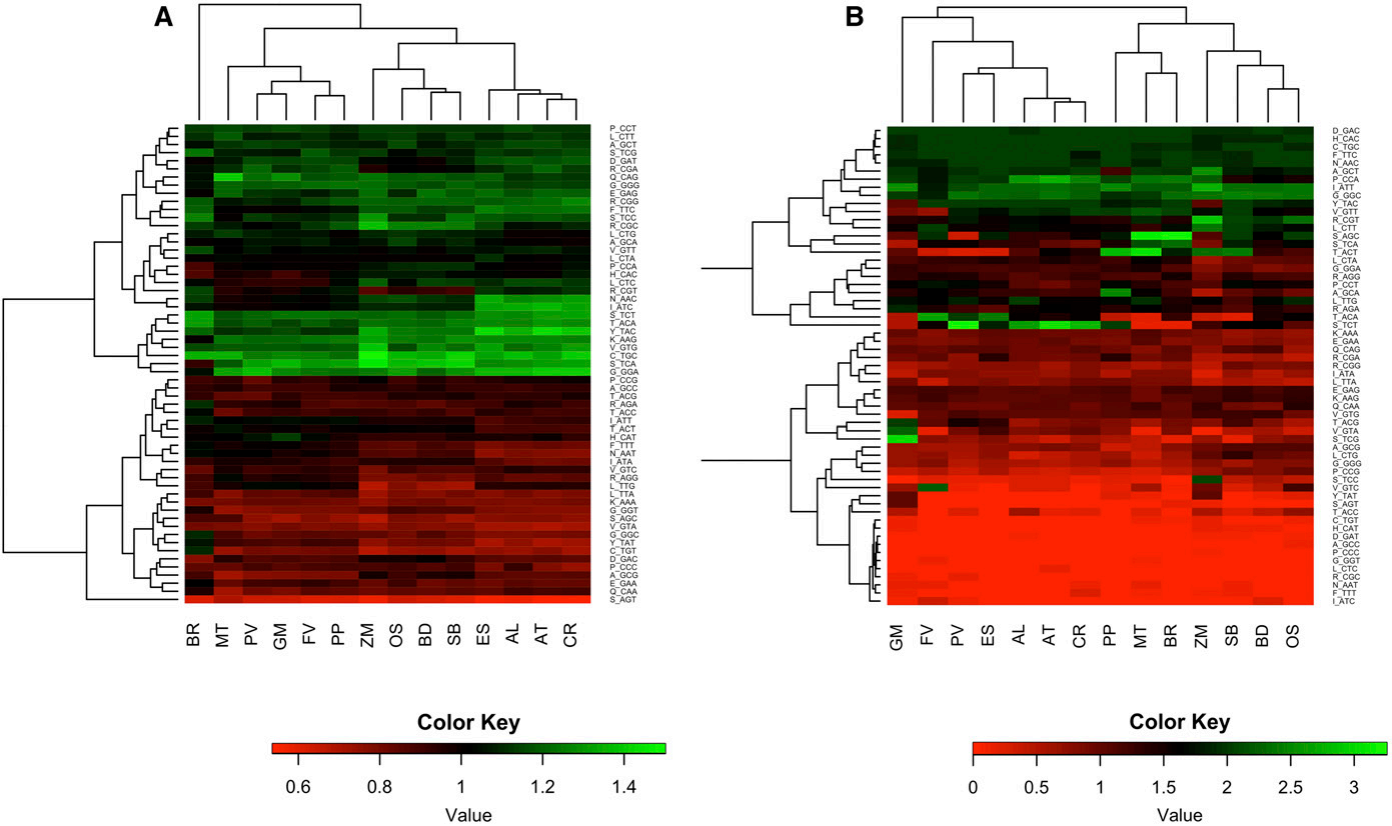
(A) Heat map showing the odds ratios for each codon in each species based on Seforta codon enrichment analysis (the average of all pairwise comparisons within each clade). (B) Heat map showing the tRNA-RSCU values in each species.

This analysis revealed several codons preferentially found in the conserved sites (Figure 2A and Figure S1), thus identifying a potential set of accurate codons. Because the evolutionary state of each site was inferred based on changes in related lineages, the effects of differences in the rates and/or direction of adaptive amino acid substitutions among lineages could be raised as a concern. We found a consistent pattern of potential accurate codons regardless of the sister species used to infer unconstrained sites. This suggests that the eventual species-dependent differences in nonsynonymous changes had a limited impact on our analysis. We also compared datasets (see *Materials and Methods*) that were obtained by the pairwise alignment of orthologous genes (original Akashi test) with those obtained with the phylogeny-based approach (phylogeny-based Akashi test). Notably, all amino acid changes are considered in the former approach regardless of the lineage in which they occurred. The odds ratios calculated using both approaches were strongly correlated (MH test, *r* = 0.82; *P* < 0.0001), again confirming the robustness of our phylogeny-driven approach.

In general, codons at conserved sites proved to be strongly GC-rich at the third base position in monocots, with a similar but less extreme trend observed in dicots and even the opposite trend in some legumes (Table 1 and Table S3). The preferential use of particular sets of synonymous codons at conserved sites did not emerge in all species with the same intensity. Indeed, we plotted the average deviation (absolute value) of all the calculated odds ratios from the neutral value 1 and found that the fabids were characterized by significantly lower values than the malvids, whereas the monocots featured intermediate values (Figure S2). The fabids were characterized by the most homogenous trends within each family whereas more variability was observed for monocots and malvids, with *B. rapa* yielding the lowest values of all the species in its clade.

**Table 1:** Average GC3 content of optimal and accurate codons in four species belonging to the fabids, malvids, and monocots

Species	Average GC3	
	Optimal Codons	Accurate Codons
*A. thaliana*	67.9	55.2
*M. truncatula*	70.8	44.8
*O. sativa* HGC	73.7	100.0
*O. sativa* LGC	40.0	51.9
*Z. mays* HGC	17.7	95.5
*Z. mays* LGC	6.3	50.0

Similar odds ratios were observed for certain codons in all the species we investigated, including TGC, GTG, and TCT. However, others showed clade-dependent behavior, *e.g.*, ATC and AAC were strongly overrepresented at conserved sites in the Brassicaceae whereas CGC was overrepresented at conserved sites only in monocots. Certain motifs occurred frequently in the underrepresented codons, including the dinucleotides GT and TA at the second and third codon position for all species. In general, the preference for specific codons at conserved sites proved to be evolutionarily conserved, with fabids, malvids, and monocots clustering in three distinct groups, and *B. rapa* as the only exception (Figure 2A).

If there is a relationship between the abundance of aminoacyl-tRNAs and the time taken to occupy the acceptor site on the ribosome, the codons corresponding to abundant tRNAs are likely to be translocated faster than other codons (Sharp and Li 1987). Because aminoacyl-tRNAs compete at the ribosome acceptor site until the correct one is stably installed, abundant tRNAs (and the corresponding codons) may therefore be associated with a lower mistranslation rate. We used the number of tRNA gene copies found using the tRNAscan tool as a proxy to establish the most abundant isoacceptors in the cytosol. Our results (Figure 2B) did not reveal a clear association between codons featuring the most abundant isoacceptors within each synonymous group (tRNA-RSCU) and the preference at codons at conserved sites. However, the relative number of tRNA gene copies has been conserved during evolution and the three plant families partially cluster when the tRNA-RSCU values are used as variables. We ran an MH test on the two matrices used for the cluster analyses presented in Figure 2, A and B (odds ratios and tRNA-RSCU values) to verify the degree of association, resulting in a correlation of 0.279. This value was nonsignificant (*P* = 0.058 following 100,000 completely random permutations; *P* = 0.06 when permutating only the corresponding odds ratios between species to preserve the internal correlation structure) but it revealed a potential relationship between these two sets of variables. Indeed, several species showed a significant correlation between the odds ratio and tRNA-RSCU value for each codon (Table S4).

### Codon usage bias and the protein domain effect

**Figure 3:**
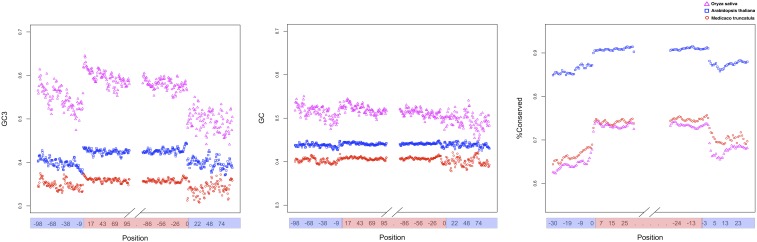
Average GC3, average GC, and percentage of conserved sites within different portions of the transcripts in *A. thaliana*, *M. truncatula*, and *O. sativa*. Position axes refer to the number of sliding windows analyzed (window step = 3 nt/1 amino acid; window size = 9 nt/3 amino acids). Average GC3 and GC values are calculated at a nucleotide level, whereas the percentage of conserved sites refers to the protein coordinates. Red represents the domain regions and blue represents the nondomain regions.

If the abundance of tRNAs cannot completely account for codon usage bias at evolutionarily conserved sites, additional factors other than accuracy should be taken into consideration. The portions of mRNAs encoding protein domains are significantly more structured than those specifying interdomain regions in *A. thaliana*, possibly because selection slows down the ribosomal machinery to ensure the correct folding of functionally critical protein regions (Vandivier *et al.* 2014; Homma *et al.* 2016). To visualize the compositional bias at domain and interdomain regions, we analyzed transcript portions representing protein domains as well as the corresponding nondomain flanking regions. Both the GC3 value and, to lesser extent, the GC content proved to be higher in the domain sequences (Figure 3 and Table 2). We also observed a higher number of evolutionarily constrained sites preferentially located within domain regions (Figure 3). Taken together, such observations suggest that the nucleotide composition within domain regions can significantly influence the codon usage bias.

**Table 2:** Compositional statistics of four plant species belonging to the three clades (*A. thaliana* for malvids, *M. truncatula* for fabids, and *O. sativa* and *Z. mays* for monocots)

				Odds Ratio				
Species	ΔGC3 (D−ND)	Δ%Conserved (D−ND)	Δ%Conserved (S−L)	All	Domains	Nondomains	Stems	Loops
*A. thaliana*	2.8	5.29	0.40	1.11	1.14	1.09	1.10	1.14
*M. truncatula*	1.5	14.97	1.21	1.03	1.07	1.02 ns	0.99 ns	1.03 ns
*O. sativa* HGC	7.4	19.27	0.03 ns	1.08	1.01 ns	1.06	1.02 ns	1.13
*O. sativa* LGC	2.9	17.64	0.26 ns	1.06	1.02 ns	1.01 ns	1.07	1.06
*Z. mays* HGC	5.1	18.15	0.28 ns	0.79	0.84	0.79	0.80	0.78
*Z. mays* LGC	1.0	16.34	0.38 ns	1.05	1.00 ns	1.02 ns	1.00 ns	1.06

Odds ratios refer to the Akashi test on the genomes of each species calculated for: all, overall transcripts; domains, transcript segments covering protein domain regions; nondomains, transcript segments covering nondomain regions; stems, transcript portions containing codons with the third base in a stem region; and loops, transcript portions containing codons with the third base in a loop region. D, codons in protein domain regions; ND, codons in protein nondomain regions; S, codons with the third base in a stem region; L, codons with the third base in a loop region; ns, nonsignificant.

Codon enrichment within domain sequences (MH test of domain *vs.* nondomain regions) and at conserved sites (MH test of conserved *vs.* variable sites) were not strongly associated (Table S5). This observation indicates that the domain effect cannot be explained simply by the prevalence of conserved sites at these transcript regions. We therefore separated the effects resulting from evolutionary conservation or inclusion within a protein domain. Codon enrichment at conserved and variable sites was studied separately in domain and nondomain regions, resulting in a strong correlation between the codon odds ratios calculated in these two datasets (Table S6). This result suggests that the evolutionary forces responsible for codon bias when comparing conserved and variable sites act upon both domain and nondomain regions of the transcript. However, the extent of codon enrichment sometimes differed quantitatively between the two regions (Figure S3 and Table S7, a and b), suggesting that codon choice at each site could also depend on its inclusion in or exclusion from a functional protein domain.

Further insight was gained by analyzing codon enrichment at conserved sites within either domain or nondomain regions. If the domain plays a role in codon choice then there should be a difference in codon bias between these datasets, which was indeed the case. Strong differences were especially prevalent in monocots, with codons ending in GC preferentially used at conserved sites within the domain regions of HGC genes and the nondomain regions of LGC genes (Figure S4a and Table S7c). Several reports have described intragenic compositional patterns within plant genes (Wong *et al.* 2002; Porceddu and Camiolo 2011; Ressayre *et al.* 2015). For example, the GC content at the third codon base decreases along the direction of transcription in monocot genes (Wong *et al.* 2002; Porceddu and Camiolo 2011). It is possible that this effect could influence our analysis if constrained/domain sites are not uniformly distributed along the CDS. To measure the potential impact of this phenomenon, we divided each transcript into three portions and repeated the analysis separately on each portion, using *A. thaliana* and *O. sativa* to represent dicots and monocots, respectively. As shown in Figure S5, the results from the entire CDS were generally confirmed in each separate coding portion. The only exception were the nondomain LGC rice genes, where the observed trend was mainly concentrated in the first portions of the transcripts.

### Codon usage bias and mRNA structural constraints

Secondary structures in mRNAs can also form outside the regions of the transcript corresponding to protein domains (Lorenz *et al.* 2011) and regulatory structures may be present in other genic portions (Kirby *et al.* 1995; Ringnér and Krogh 2005), although whether such motifs also occur in CDS remains unclear due to potential functional constraints. Recently, mature mRNA was shown to be under selection for secondary structure in yeast, and the third base of the codon is under selection to maintain mRNA packing, a structural constraint that has a minor impact compared to that observed in the untranslated regions (Stoletzki 2008). Accordingly, we used RNAfold to calculate the most probable secondary structures of transcripts in our investigation and identified codons with an inferred strongly paired third position, *e.g.*, those included in stem regions.

Codon enrichment analysis revealed that several codons are used preferentially in stems compared to loops and can be considered as structurally preferred codons (Table S5). Interestingly, we found that codon preferences in stems and at evolutionarily conserved sites were only marginally associated. Indeed, codons preferentially found in stems are not always the most frequent at evolutionarily conserved sites, and as anticipated, usually end in GC (Table S5). Furthermore, the stem regions are only slightly enriched in evolutionarily conserved sites compared to loops, and the percentage variation in conserved sites is significant only in dicots (Table 2). These findings suggest that synonymous codon choice at a given conserved site may also depend, albeit marginally, on the stem/loop state. To test this hypothesis, we analyzed codon preferences between conserved and variable sites, looking separately at stem and loop regions, and codon usage bias was observed in both datasets. Interestingly, the relationship between codon usage preference and the evolutionary characteristics of each site was similar in the two datasets (Figure S6 and Table S7, d and e), although codons ending in GC often had a lower odds ratio in stems than loops. We also investigated the effect of structure by studying the relationship between codon choice at conserved sites and their stem/loop state. We found that conserved sites in stems tended to be slightly enriched for codons ending in GC and more strongly enriched for codons ending in AG (Figure S4b). These results suggest that structural selection has little if any impact on the detection of signatures of translational accuracy, although it cannot be ruled out altogether.

### Balancing translational accuracy, efficiency, and nonsynonymous mutation

Selection can maximize the accuracy and efficiency of protein synthesis simultaneously if the synonymous codons that are most accurately translated are also those that are served more quickly by the corresponding tRNAs (Akashi 1994). Accordingly, codons that are preferred in terms of translational efficiency are also more abundant in *D. melanogaster* (Akashi 1994), plants (Porceddu *et al.* 2013), humans (Drummond and Wilke 2008), yeast (Zhou *et al.* 2009), and nematodes (Marais and Duret 2001). We confirmed above that codon bias at a given site in plants is influenced not only by its evolutionary state but also by its position with respect to protein domains and mRNA secondary structures. We therefore set out to investigate the balance between translational accuracy and efficiency.

We first determined the optimal codons by statistical analysis of the differences in synonymous codon usage between strongly and weakly expressed genes (Table S5). Monocot gene sets were split into HCG and LCG subsets to account for differential selection pressures that may influence these two groups of genes (Mukhopadhyay *et al.* 2008). Optimal codons generally proved to be GC-rich at the third base but, exceptionally, the optimal codons in *O. sativa* LGC genes and all *Z. mays* genes proved to be strongly AT-rich (Table 1). Notably, the optimal codons we determined in *Z. mays* differed significantly from those previously reported based on the analysis of correspondence (COA) (Liu *et al.* 2010). Indeed, whereas we directly compared the codon usage in strongly and weakly expressed genes, the COA approach investigates codons that are used in the most biased genes, which are typically expressed at higher than average levels. A correlation analysis between the codon bias (calculated by the mean effective number of codons) and their expression in *Z. mays* revealed a significant positive association between these two parameters (Pearson *r* = 0.38, *P* < 0.0001). In other words, the most biased genes are those expressed at the lowest levels. The MH test was carried out to investigate the use of optimal codons at conserved *vs.* variable sites, *i.e.*, the Akashi test (Akashi 1994), using Seforta on the *A. thaliana*, *M. truncatula*, *O. sativa*, and *Z. mays* datasets. This confirmed the low odds ratio in plants and revealed only moderate signatures of translational accuracy in these species. The highest values were observed in *A. thaliana* and *O. sativa*, and the lowest values were observed in the legume *M. truncatula*. Interestingly, *Z. mays* HGC genes yielded a significant odds ratio but the value was negative, which predicts that optimal codons are preferentially used at nonconserved sites rather than conserved sites.

It is possible that nonsynonymous mutations significantly bias the measured effect of selection for translational accuracy. For example, the amino acid change from glycine to alanine following the single base mutation GTA → ATA, may replace the favored codon with a disfavored one or vice versa. To gain insight into the balance between selection for translational accuracy and nonsynonymous mutation, we constructed an additional dataset that included all sites that conserved their condition with regard to selection (both favored or both disfavored) despite the amino acid change. In practice, we considered only variable sites in which the two codons kept the same favored (third) base and the same optimality (optimality-tied Akashi test). Amino acid changes that involved first and second codon base mutations were considered only if all intermediate states in all reconstructed mutational pathways included the same potential silent position bases and the same favored silent bases. As shown in Table S8, the results obtained with the whole dataset and the optimality-tied dataset were very similar, suggesting that nonsynonymous mutations have a negligible impact on codon usage bias.

As previously observed, several factors may influence codon usage, such as selection for mRNA stability in transcript regions corresponding to proteins domains (Vandivier *et al.* 2014) or mRNA secondary structures (Stoletzki 2008). To determine the effects of these factors on translational accuracy, we split the CDS into two datasets representing codons in domain and nondomain regions, and signatures for the selection of translational accuracy were sought for these new datasets by applying separate Akashi tests. In *A. thaliana*, signatures of selection for translational accuracy were found in both the domain and nondomain regions, with odds ratios that were slightly higher in the domain region dataset. A similar picture emerged in *M. truncatula*, although the odds ratio was not significant in the nondomain regions. Interestingly, signatures of translational accuracy initially detected in the complete monocot LGC datasets disappeared when the domain and nondomain regions were analyzed separately, whereas a significant odds ratio was calculated for the nondomain regions dataset of *O. sativa* HGC genes, and negative values were recorded once again for both the domain and nondomain regions datasets of *Z. mays* HGC genes.

In order to control for the selection of mRNA secondary structures, we split the CDS into two datasets representing codons featuring a strongly paired third base (stems) or a weakly paired/nonpaired third base (loops). Again, separate Akashi tests were carried out on each dataset to find signatures for the selection of translational accuracy while controlling for the effect of stem/loop structure. We generally observed slightly higher odds ratios when only codons in loop regions were tested, but in many cases, there was no substantial difference between codons in stems and loops (Table 2 and Table S8).

### Variation of translational accuracy along the transcript

Compositional gradients have been widely documented in plant mRNAs (Porceddu and Camiolo 2011; Ahmad *et al.* 2013). To determine whether this phenomenon is associated with signals of selection for translational accuracy, we split each alignment using an adjacent window approach and looked for gradients of codon enrichment. Variation might also be associated with differential selective pressure for translational efficiency in the direction of the CDS, so we also calculated the Fop along the transcript. *A. thaliana*, *M. truncatula*, and *O. sativa* were selected as representatives of the malvids, fabids, and monocots, respectively.

**Figure 4:**
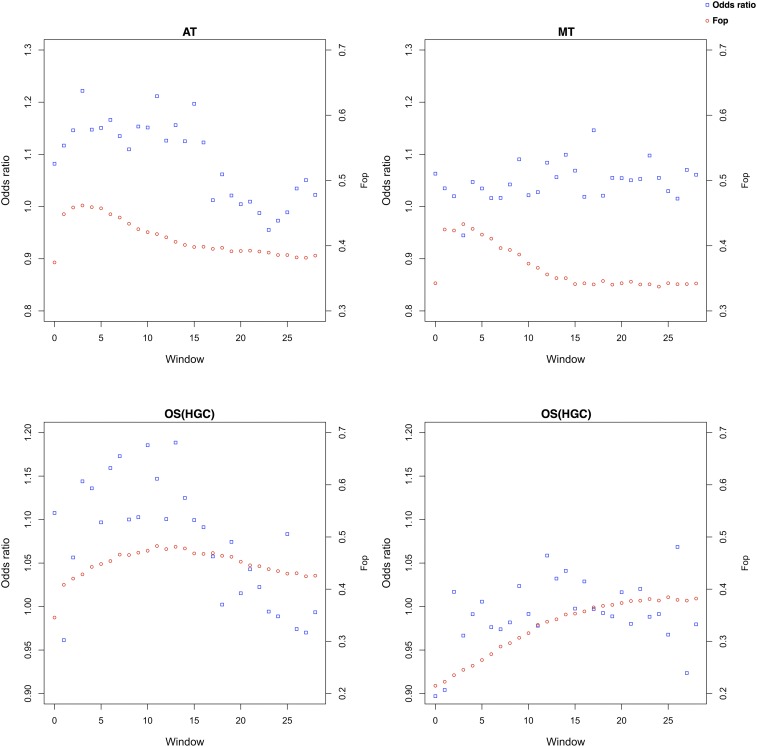
Variation of Fop and translational accuracy along the transcript. Each value represents an adjacent window containing five amino acids. The corresponding codons were pooled from all the genes to form a supersequence, which has been used for the calculations.

In *A. thaliana*, we found that signals for the selection of translational accuracy were stronger in the 5′ portion of the transcript and declined beyond the first 75 codons. Furthermore, the Fop index in this species initially increased and then declined along the CDS, suggesting there is a differential selection pressure on the first portion of the CDS (Figure 4). Low values of translational accuracy along the transcript were observed in *M. truncatula*, although the Fop index showed the same peak then decline behavior observed in *A. thaliana*. A variation in the calculated odds ratio was observed in monocots, with *O. sativa* HGC genes revealing a pattern similar to *A. thaliana*, whereas *O. sativa* LGC genes showed considerable variability, with only few regions revealing a signal for the selection of translational accuracy.

## Discussion

### Codon composition at conserved sites

Selection for translational accuracy is a widely studied phenomenon and identification relies on several assumptions (Akashi 1994) that, we believe, deserve careful investigation. Indeed, for amino acid changes due to single base substitutions (*e.g.*, the first or second codon base), the base at the third codon position is (initially) determined by the wild-type third codon position. This effect is particularly relevant for young changes for which mutation/selection had limited opportunities to reestablish the appropriate favored/unfavored third base. We controlled for such effects by limiting the analysis to unconstrained sites that changed in closely related lineages. However, the unique evolutionary histories of each species and their phylogenetic distances may influence the validity of these assumptions. For example, if protein evolution is driven mainly by adaptive selection then the simple equivalence between evolutionary conservation and structural/functional importance may be inappropriate. We attempted to account for these effects by considering each species in relation to several sister species. In addition, we demonstrated that the results obtained by considering all changed sites regardless of the lineage in which they occurred did not change the picture substantially.

We investigated the signatures of translational accuracy in three different plant clades (fabids, malvids, and monocots) to gain insight into the evolution of this phenomenon. We started by analyzing the synonymous codon composition at conserved sites using a phylogeny-based approach within each clade, in order to estimate the variability of the results. This revealed that synonymous codon preference at conserved sites is similar within clades and can be used to assign each species to its phylogenetic group.

Interestingly, such accurate codons are not always the same as the optimal codons, *i.e.*, those used in the most strongly expressed genes or matching the most abundant tRNAs (Table S5). This agrees with the Akashi test results, yielding values only marginally >1 for each of the plant species we investigated (Table 2 and Table S8), and contrasts with previous studies in other organisms (Drummond and Wilke 2008). Signatures of translational accuracy are evident in plants not only from the Akashi test results but also by considering the recurrence of certain dinucleotide motifs in these accurate codons. For example, codons containing the notably counterselected dinucleotides TA, GT, or CG at the second and third positions (Porceddu and Camiolo 2011) were generally underrepresented. Notably, the weak signal of translational accuracy in plants should be considered in the context of its variability along the transcript. Indeed, even the legume genomes, which tended to feature minimal signals of translational accuracy, showed evidence of such signals at the beginning of the CDS in line with observations in other eukaryotes.

We considered what mechanisms might influence the enrichment of certain synonymous codons at conserved sites when they do not mirror the most abundant tRNAs. Similarly, the apparently anomalous odds ratio values observed in *Z. mays* also called for further investigation. Our evidence suggests that the measurement of signatures of selection for translational accuracy requires the investigation of several different selective forces, some of which are conserved at the clade or species level.

### Structural determinants

We found that conserved sites are preferentially located in CDS regions corresponding to functional domains, whose GC3 enrichment may promote secondary structures that can delay the translational machinery and thus facilitate correct folding in these critical regions (Vandivier *et al.* 2014). This may influence the detection of signatures of translational accuracy because optimal codons in plants are generally GC-rich at the third codon position (Table 1), and may therefore be used preferentially at conserved sites only because these are prevalent within protein domains. We investigated this hypothesis by splitting the datasets into domain and nondomain regions because any differences reflecting the concentration of biased conserved sites in domains should disappear when the two CDS portions are analyzed separately. In *A. thaliana* and *M. truncatula*, the signature of translational accuracy proved to be even stronger within protein domains (Table 2 and Table S8). This confirmed that selection for translational accuracy occurs in these species because the incorporation of inappropriate tRNAs is likely to be counterselected, especially in functionally or structurally critical portions such as domains. The opposite trend was observed in monocots, in which signatures of translational accuracy can be totally explained by the differential distribution of conserved sites between domain and nondomain regions, particularly in LGC genes. This evidence, combined with the independent Akashi test responses along the transcript (Figure 4), indicates the absence of a true signature of selection for translational accuracy in *O. sativa* LGC genes. The observed results may reflect the nonuniform distribution of domain regions within the transcripts, so we also repeated our analysis by splitting the CDS into three portions and performing the Akashi test separately on each. The results were generally in agreement with those based on the entire transcript.

Although the structural constraint of domain regions influences synonymous codon choice at conserved sites in some species, we propose that selection for translational accuracy can, in most cases, have an impact on the CDS. Indeed, we found that optimal codons ending in AT tended to be overrepresented at conserved sites in domain regions compared to nondomain regions, despite the general GC enrichment in the domain regions of most of the species we investigated (Figure S3). Only HGC genes in monocots showed the opposing trend, *i.e.*, optimal codons ending in AT tended to be underrepresented in the domain regions (Figure S3). We calculated the difference between the odds ratios of each codon at conserved and variable sites in domain and nondomain regions (Figure S3), and studied its variation with the odds ratio that emerged from the computation of the optimal codons. This revealed a significant correlation between the two variables for most of the species included in this investigation (Table S9), indicating that conserved sites in domains are translated more accurately than conserved sites in nondomain regions.

We also controlled for the enrichment of mRNA stem and loop structures at conserved sites and found significant differences in distribution among the dicots but not the monocots. These results should be considered critically because although RNAfold predicts the most likely RNA secondary structures, empirical data indicate only a small (albeit significant) correlation between the predictions and experimentally determined stabilities (Stoletzki 2008). Interestingly, the signal of translational accuracy disappeared in the legume *M. truncatula* when stem and loop regions were analyzed separately. This suggests that selection for mRNA secondary structure may prevent the correct detection of signatures of translational accuracy. However, translational accuracy in *M. truncatula* was found to be low compared to the whole dataset, and the diminished odds ratios together with the loss of significance may reflect the smaller codon sample size in the separate stem and loop datasets. The general lower odds ratios found in stem regions also suggests there is selection pressure at sites critical for correct folding, *i.e.*, selection for translational accuracy is more likely to affect loop regions that are not under structural selection.

The structural constraints driving mRNA folding may influence codon usage at conserved sites. Indeed, conserved sites in stems were preferentially represented by codons ending in GC compared to conserved sites in loops (Figure S5b). However, this effect is concomitant with the anticipated preferential use of optimal codons ending in AT in stem regions, with translational accuracy dominating the stem/loop effect. Interestingly, the codons most strongly overrepresented in stems compared to loops were those ending in AG, indicating that selection pressure may act to sequester this dinucleotide in stem regions to minimize additional splicing phenomena, as recently reported in *A. thaliana* (Staiger and Simpson 2015).

### Genome evolution

We found that the codon sets at codons at conserved sites were generally conserved within each clade, but *B. rapa* was exceptional, probably reflecting the unique evolutionary history of this species. *B. rapa* has experienced a recent whole genome triplication event and the three resulting subgenomes have evolved independently, *i.e.*, the fractionation rate differs among these three genomic portions (Milia *et al.* 2015; Cheng *et al.* 2012).

Genome evolution may also explain the results we observed in *Z. mays*, *i.e.*, the significant negative odds ratio. Monocot genomes experienced a rapid increase in GC content following their separation from the common ancestor of monocots and dicots (Clement *et al.* 2015). This increase, together with the compositional gradients along transcripts, may have resulted in the emergence of HGC genes. However, *Z. mays* features by far the largest genome among the plants we analyzed, and processes such as replication and genome repair are more of a burden on resources when the GC content is particularly high (Šmarda *et al.* 2014). This species may therefore be under more selection pressure to reduce the GC content than the other monocots we investigated, with the erosion more evident in GC-rich genes (Clement *et al.* 2015). Indeed, *Z. mays* was the only species featuring AT3-rich optimal codons. Because conserved residues are preferentially found in domain regions that tend to be GC-rich for structural reasons, the AT3-rich optimal codons are more likely to be used in nondomain regions and variable CDS regions, which would explain the negative odds ratio we observed.

### The impact of selection

We observed a difference in the strength of the translational accuracy signal between *A. thaliana* and the legume *M. truncatula*, with the latter showing lower odds ratios (potentially influenced by structural selection as described above) that may indicate more relaxed selection. We recently reported that general genomic enrichment for AT in dicots is balanced by selection-driven enrichment for GC in CDS (Camiolo *et al.* 2015). Legumes have the most AT-rich genome, further supporting the more relaxed selection pressure on this species (Camiolo *et al.* 2015). Indeed, when investigating the main differences in synonymous codon usage at conserved sites, the presence of several codons ending in TC distinguished the legumes from the other species we analyzed (Figure S1). This dinucleotide is a known mutation hotspot because the deamination of a methylated cytosine would give rise to TT, and it was suppressed in the conserved sites of all the species we analyzed except the legumes.

In contrast, the stronger effect of selection on *B. rapa* CDS may help to explain the differences between this species and the remaining Brassicaceae. A comparison of *B. rapa* and *A. thaliana* transcripts showed that a higher proportion of optimal codons are used in *B. rapa* CDS, possibly because outcrossing provides more genomic diversity for selection (Tiffin and Hahn 2002). The higher population size of *B. rapa* may have also hindered the fixation of alterative alleles due to drift, and enhanced the strength of both purifying and positive selection as previously reported for other Brassicaceae species (Slotte *et al.* 2010). Moreover, the higher recombination in allogamous species may strengthen the effect of selection, possibly due to a reduced Hill–Robertson effect (Comeron and Guthrie 2005). Taken together, such evidence may result in an increased number of optimal codons along the transcript, thus flattening the compositional differences between conserved and variable sites.

### Physiological and ecological factors

Clade-specific codon usage at conserved sites and similar trends in the Akashi test results may also have a physiological basis. For example, the weak signal for the selection of translational accuracy in legumes may reflect the ability of legumes to fix nitrogen, which could offset the costs of mistranslation and permit the relaxation of selection pressure. The opposite trends in *O. sativa* and *Z. mays* may be associated with the ecology of these plants. The higher GC content of monocot genomes and the emergence of HGC genes have been proposed as mechanisms to deal more effectively with biotic and abiotic stress (Šmarda *et al.* 2014). *O. sativa* is cultivated under diverse ecological conditions and can deal with more biotic stress conditions than *Z. mays*, which may explain why the erosion of the GC content in *Z. mays* HGC genes is not counterselected to maintain this class of genes.

## Supplementary Material

Supplemental material is available online at www.g3journal.org/lookup/suppl/doi:10.1534/g3.117.040626/-/DC1.
