# Supplementary material for: The Evolutionary Basis of Translational Accuracy in Plants
Source: G3 (Bethesda). 2017 May 22;7(7):2363–73. doi: 10.1534/g3.117.040626 (PMC5499143; doi:10.1534/g3.117.040626)
Supplement: Supplementary file 1 [file 2363TableS1.docx]

**Table S1:** List of the microrarray experiments that were used to estimate the expression levels of genes in the following species.

| **Species** | **http** |
| --- | --- |
| A thaliana | <http://www.plexdb.org/modules/PD_browse/experiment_browser.php?experiment=AT40> |
| M truncatula | <http://www.plexdb.org/modules/PD_browse/experiment_browser.php?experiment=ME1> |
| O sativa | <http://www.plexdb.org/modules/PD_browse/experiment_browser.php?experiment=OS5> |
| Z. mays | <http://www.ncbi.nlm.nih.gov/geo/query/acc.cgi?acc=GSE27004> |
