# Supplementary material for: The Evolutionary Basis of Translational Accuracy in Plants
Source: G3 (Bethesda). 2017 May 22;7(7):2363–73. doi: 10.1534/g3.117.040626 (PMC5499143; doi:10.1534/g3.117.040626)
Supplement: Supplementary file 2 [file 2363TableS2.docx]

**Table S2:** The table reports the species triplets that have been used in the parsimony-based Akashi test.

| **Species under study** | **Sister species** | **Outgroup** |  | **Species under study** | **Sister species** | **Outgroup** |
| --- | --- | --- | --- | --- | --- | --- |
| AL | AT | ES |  | GM | PP | FV |
| AL | BR | ES |  | GM | PV | PP |
| AL | CR | ES |  | MT | FV | PP |
| AL | ES | BR |  | MT | GM | PP |
| AT | AL | ES |  | MT | PP | FV |
| AT | BR | ES |  | MT | PV | PP |
| AT | CR | ES |  | PP | FV | MT |
| AT | ES | BR |  | PP | GM | MT |
| BR | AL | AT |  | PP | MT | GM |
| BR | AT | AL |  | PP | PV | MT |
| BR | CR | AT |  | PV | FV | PP |
| BR | ES | AT |  | PV | MT | PP |
| CR | AL | ES |  | PV | GM | FV |
| CR | AT | ES |  | PV | PP | PP |
| CR | BR | AT |  | BD | OS | SB |
| CR | ES | AT |  | BD | SB | ZM |
| ES | AL | AT |  | BD | ZM | SB |
| ES | AT | AL |  | OS | BD | SB |
| ES | BR | AT |  | OS | SB | ZM |
| ES | CR | AT |  | OS | ZM | SB |
| FV | GM | MT |  | SB | BD | OS |
| FV | MT | GM |  | SB | OS | BD |
| FV | PP | MT |  | SB | ZM | OS |
| FV | PV | MT |  | ZM | BD | OS |
| GM | FV | PP |  | ZM | OS | BD |
| GM | MT | PP |  | ZM | SB | OS |
