# Supplementary material for: The Evolutionary Basis of Translational Accuracy in Plants
Source: G3 (Bethesda). 2017 May 22;7(7):2363–73. doi: 10.1534/g3.117.040626 (PMC5499143; doi:10.1534/g3.117.040626)
Supplement: Supplementary file 3 [file 2363TableS3.docx]

**Table S3:** Average GC3 content of the accurate codons for each species.

| Species | % GC3 in accurate codons |
| --- | --- |
| AL | 57.1 |
| AT | 55.2 |
| BR | 50.0 |
| CR | 55.2 |
| ES | 54.8 |
| FV | 51.6 |
| GM | 51.9 |
| MT | 44.8 |
| PP | 53.3 |
| PV | 44.8 |
| BD | 66.7 |
| OS | 58.6 |
| SB | 56.7 |
| ZM | 58.6 |
