# Supplementary material for: The Evolutionary Basis of Translational Accuracy in Plants
Source: G3 (Bethesda). 2017 May 22;7(7):2363–73. doi: 10.1534/g3.117.040626 (PMC5499143; doi:10.1534/g3.117.040626)
Supplement: Supplementary file 4 [file 2363TableS4.docx]

| **Species** | **Spearman ρ** |
| --- | --- |
| **AL** | 0.32* |
| **AT** | 0.34* |
| **BD** | 0.31* |
| **BR** | 0.04 |
| **CR** | 0.30 |
| **ES** | 0.31 |
| **FV** | 0.29* |
| **GM** | 0.03 |
| **MT** | 0.18 |
| **OS** | 0.25 |
| **PP** | 0.24 |
| **PV** | 0.32* |
| **SB** | 0.24 |
| **ZM** | 0.15 |

**Table S4:** Spearman correlation between the odds ratios and the tRNA-RSCU values of 59 codons for each species (* p<0.05).
