# Supplementary material for: The Evolutionary Basis of Translational Accuracy in Plants
Source: G3 (Bethesda). 2017 May 22;7(7):2363–73. doi: 10.1534/g3.117.040626 (PMC5499143; doi:10.1534/g3.117.040626)
Supplement: Supplementary file 5 [file 2363TableS5.docx]

**Table S5:** Codon enrichment analysis of several datasets for five plant species belonging the malvids (AT), fabids (MT and GM) and monocots (OS and ZM). D = codon enrichment in protein regions corresponding to domains vs non-domain regions. S = codon enrichment in transcript regions corresponding to stems vs loops, A = codon enrichment in evolutionarily-conserved vs variable sites (accurate codons), O = codon enrichment in strongly vs weakly expressed genes (20%, optimal codons), T = codons with tRNA-RSCU values >1.

|  | **AT** | | | | |  | **MT** | | | | |  | **GM** | | | | |  | **OS (HGC)** | | | | |  | **OS (LGC)** | | | | |  | **ZM (HGC)** | | | | |  | **ZM (LGC)** | | | | |
| --- | --- | --- | --- | --- | --- | --- | --- | --- | --- | --- | --- | --- | --- | --- | --- | --- | --- | --- | --- | --- | --- | --- | --- | --- | --- | --- | --- | --- | --- | --- | --- | --- | --- | --- | --- | --- | --- | --- | --- | --- | --- |
| **AA_Codon** | **D** | **S** | **A** | **O** | **T** |  | **D** | **S** | **A** | **O** | **T** |  | **D** | **S** | **A** | **O** | **T** |  | **D** | **S** | **A** | **O** | **T** |  | **D** | **S** | **A** | **O** | **T** |  | **D** | **S** | **A** | **O** | **T** |  | **D** | **S** | **A** | **O** | **T** |
| A_GCA |  |  |  |  | X |  |  |  | X |  | X |  | X |  | X |  | X |  |  |  | X |  |  |  | X |  | X |  |  |  |  |  | X | X |  |  | X |  | X | X |  |
| A_GCC |  | X |  | X |  |  |  | X |  | X |  |  |  | X |  | X |  |  | X |  |  | X |  |  |  | X |  |  |  |  | X |  |  | X |  |  |  | X |  |  |  |
| A_GCG |  | X |  |  |  |  |  | X |  |  |  |  |  | X |  |  |  |  |  | X |  |  | X |  |  | X |  |  | X |  | X | X |  |  |  |  |  | X |  |  |  |
| A_GCT |  | X | X | X | X |  |  | X | X | X | X |  | X | X | X | X | X |  |  |  | X |  | X |  | X |  | X | X | X |  |  |  | X | X | X |  | X |  | X | X | X |
| C_TGC | X | X | X | X | X |  |  | X | X |  | X |  |  | X | X | X | X |  | X | X | X | X | X |  |  | X | X |  | X |  | X | X | X |  | X |  |  | X | X |  | X |
| C_TGT |  |  |  |  |  |  |  |  |  |  |  |  |  |  |  |  |  |  |  |  |  |  |  |  | X |  |  |  |  |  |  |  |  | X |  |  | X |  |  | X |  |
| D_GAC | X | X |  | X | X |  | X |  |  | X | X |  |  | X |  |  | X |  | X | X |  |  | X |  |  |  |  |  | X |  | X | X |  |  | X |  |  | X |  |  | X |
| D_GAT |  |  | X |  |  |  |  |  | X |  |  |  | X |  | X |  |  |  |  |  | X |  |  |  | X |  | X | X |  |  |  |  | X | X |  |  | X |  | X | X |  |
| E_GAA |  |  |  |  |  |  |  |  |  |  | X |  |  |  |  |  |  |  |  |  |  |  |  |  | X |  |  |  |  |  |  |  |  | X |  |  | X |  |  | X |  |
| E_GAG | X | X | X | X |  |  | X | X | X | X |  |  | X | X | X | X | X |  | X | X | X | X | X |  |  | X | X | X | X |  | X | X | X |  | X |  |  | X | X |  | X |
| F_TTC |  | X | X | X | X |  |  | X | X | X | X |  |  | X |  |  | X |  | X | X | X | X | X |  |  | X | X |  | X |  | X | X | X |  | X |  |  | X | X |  | X |
| F_TTT |  |  |  |  |  |  |  |  |  |  |  |  | X |  |  |  |  |  |  |  |  |  |  |  | X |  |  |  |  |  |  |  |  | X |  |  | X |  |  | X |  |
| G_GGA |  |  | X |  | X |  | X |  | X | X | X |  | X |  | X |  | X |  |  |  | X |  |  |  | X |  | X |  |  |  |  |  | X | X |  |  | X |  | X | X |  |
| G_GGC |  | X |  |  | X |  |  | X |  |  | X |  |  | X |  | X | X |  | X | X |  |  | X |  |  | X |  |  | X |  | X | X |  |  | X |  |  | X |  |  | X |
| G_GGG | X | X | X |  |  |  |  | X | X |  |  |  | X | X | X |  |  |  | X | X | X |  |  |  |  | X | X |  |  |  | X | X | X |  |  |  |  | X | X |  |  |
| G_GGT |  | X |  | X |  |  |  | X |  | X |  |  |  | X |  | X |  |  |  |  |  | X |  |  | X |  |  | X |  |  |  |  |  | X |  |  | X |  |  | X |  |
| H_CAC |  |  | X | X | X |  | X |  |  | X | X |  |  |  |  |  | X |  | X | X | X | X | X |  |  |  | X |  | X |  | X | X | X |  | X |  |  |  | X |  | X |
| H_CAT |  | X |  |  |  |  |  | X | X |  |  |  | X | X | X |  |  |  |  |  |  |  |  |  | X | X |  |  |  |  |  |  |  | X |  |  | X | X |  | X |  |
| I_ATA |  |  |  |  |  |  | X |  |  |  |  |  | X |  |  |  |  |  |  |  |  |  |  |  | X |  |  |  |  |  |  |  |  |  |  |  | X |  |  |  |  |
| I_ATC |  | X | X | X |  |  |  | X |  | X |  |  |  | X |  | X |  |  | X | X | X | X |  |  |  | X | X | X |  |  | X | X | X |  |  |  |  | X | X |  |  |
| I_ATT |  | X |  |  | X |  |  | X | X |  | X |  | X | X | X | X | X |  |  |  |  |  | X |  | X | X |  | X | X |  |  |  |  | X | X |  | X | X |  | X | X |
| L_CTA |  |  | X |  | X |  | X |  | X |  | X |  | X |  |  |  | X |  |  |  |  |  | X |  | X |  |  |  | X |  |  |  |  | X |  |  | X |  |  |  |  |
| L_CTC | X | X | X | X |  |  |  | X |  | X |  |  |  | X |  | X |  |  | X |  | X | X |  |  |  | X | X |  |  |  | X |  | X |  |  |  |  | X | X |  |  |
| L_CTG |  | X |  |  |  |  |  | X | X |  |  |  |  | X | X | X |  |  | X | X | X |  |  |  |  | X | X |  |  |  | X | X | X |  | X |  |  | X | X |  | X |
| L_CTT | X |  | X | X | X |  | X |  | X | X | X |  | X |  | X | X | X |  |  |  | X | X | X |  | X |  | X | X | X |  |  |  | X | X | X |  | X |  | X | X | X |
| L_TTA |  |  |  |  |  |  |  |  |  |  |  |  |  |  |  |  |  |  |  |  |  |  |  |  | X |  |  |  |  |  |  |  |  | X |  |  | X |  |  | X |  |
| L_TTG |  | X |  |  | X |  |  | X |  |  | X |  | X | X | X |  | X |  |  | X |  |  | X |  | X | X |  |  | X |  |  | X |  | X | X |  | X | X |  | X | X |
| N_AAC | X | X | X | X | X |  |  | X |  | X | X |  |  | X | X |  | X |  | X | X | X | X | X |  |  | X | X | X | X |  | X | X | X |  | X |  |  | X | X |  | X |
| N_AAT |  |  |  |  |  |  |  |  | X |  |  |  | X |  |  | X |  |  |  |  |  |  |  |  | X |  |  |  |  |  |  |  |  | X |  |  | X |  |  | X |  |
| P_CCA |  |  | X |  | X |  | X |  |  | X | X |  | X |  |  |  | X |  |  |  | X |  | X |  | X |  | X | X | X |  |  |  | X | X | X |  | X |  | X | X | X |
| P_CCC |  |  |  | X |  |  |  |  |  | X |  |  |  |  |  | X |  |  | X |  |  | X |  |  |  |  |  |  |  |  | X |  |  | X |  |  |  |  |  |  |  |
| P_CCG |  | X |  |  |  |  |  | X |  |  |  |  |  | X |  |  |  |  |  | X |  |  |  |  |  | X |  |  |  |  | X | X |  |  |  |  |  | X |  |  |  |
| P_CCT |  |  | X | X |  |  |  |  | X |  | X |  | X | X | X | X | X |  |  |  | X |  | X |  | X | X | X | X | X |  |  |  | X | X | X |  | X | X | X | X | X |
| Q_CAA |  |  |  |  | X |  |  |  |  |  | X |  |  |  |  |  |  |  |  |  |  |  | X |  | X |  |  |  | X |  |  |  |  | X | X |  | X |  |  | X | X |
| Q_CAG | X | X | X | X |  |  | X | X | X | X |  |  | X | X | X | X |  |  | X | X | X | X |  |  |  | X | X | X |  |  | X | X | X |  |  |  |  | X | X |  |  |
| R_AGA |  |  |  |  | X |  |  |  |  |  | X |  |  |  |  |  | X |  |  |  |  |  | X |  | X |  |  |  | X |  |  |  |  | X | X |  | X |  |  | X | X |
| R_AGG |  | X |  | X | X |  |  | X | X |  | X |  | X | X |  |  | X |  |  | X |  |  | X |  |  | X |  | X | X |  |  | X |  | X |  |  |  | X |  | X |  |
| R_CGA |  |  | X |  |  |  | X |  | X |  |  |  | X |  | X | X |  |  |  |  | X |  |  |  | X |  | X |  |  |  |  |  |  |  |  |  | X |  |  |  |  |
| R_CGC | X | X | X | X |  |  | X | X | X | X |  |  |  | X | X | X |  |  | X |  | X | X |  |  |  | X | X |  |  |  | X |  | X |  |  |  |  | X | X |  |  |
| R_CGG | X | X | X |  |  |  | X | X | X |  |  |  |  | X | X |  |  |  |  | X | X |  |  |  |  | X | X |  |  |  | X | X | X |  |  |  |  | X | X |  |  |
| R_CGT | X |  | X | X | X |  | X |  |  | X | X |  | X |  |  | X | X |  |  |  |  |  | X |  | X |  |  | X | X |  |  |  |  | X | X |  | X |  |  | X | X |
| S_AGC | X | X |  | X | X |  | X | X |  | X | X |  | X | X |  |  |  |  | X | X |  |  | X |  |  | X |  |  | X |  | X | X |  |  | X |  |  | X |  |  | X |
| S_AGT | X | X |  |  |  |  | X | X |  |  |  |  | X | X |  |  |  |  |  |  |  |  |  |  | X | X |  | X |  |  |  |  |  | X |  |  | X | X |  | X |  |
| S_TCA |  |  | X |  |  |  |  |  | X |  | X |  | X |  | X |  |  |  |  |  | X |  | X |  | X |  | X |  | X |  |  |  | X | X |  |  | X |  | X | X |  |
| S_TCC |  | X | X | X |  |  |  | X |  | X |  |  |  | X | X | X |  |  | X |  | X | X |  |  |  |  | X |  |  |  | X |  | X | X | X |  |  |  | X |  | X |
| S_TCG |  | X | X | X |  |  |  | X | X |  |  |  |  | X | X |  | X |  |  | X | X |  |  |  |  | X | X |  |  |  |  | X | X |  |  |  |  | X | X |  |  |
| S_TCT |  |  | X | X | X |  |  |  | X |  |  |  |  |  | X | X |  |  |  |  | X | X | X |  | X |  | X | X | X |  |  |  | X | X | X |  | X |  | X | X | X |
| T_ACA | X |  | X |  | X |  | X |  | X |  |  |  | X |  | X |  |  |  |  |  | X |  | X |  | X |  | X |  | X |  |  |  | X | X |  |  | X |  | X | X |  |
| T_ACC |  | X |  | X |  |  |  | X |  | X |  |  |  | X |  | X |  |  | X |  |  | X |  |  |  | X |  | X |  |  | X |  |  | X |  |  |  | X |  |  |  |
| T_ACG |  | X |  |  |  |  |  | X |  |  |  |  |  | X |  |  | X |  | X | X |  |  |  |  |  | X |  |  |  |  | X | X |  |  |  |  |  | X |  |  |  |
| T_ACT |  | X |  | X | X |  |  | X | X | X | X |  | X | X |  | X |  |  |  |  |  | X | X |  | X | X |  | X | X |  |  |  | X | X | X |  | X | X | X | X | X |
| V_GTA |  |  |  |  |  |  |  |  |  |  |  |  |  |  |  |  | X |  |  |  |  |  |  |  | X |  |  |  |  |  |  |  |  | X |  |  |  |  |  |  |  |
| V_GTC |  | X |  | X |  |  |  | X |  | X |  |  |  | X |  | X |  |  | X |  |  | X | X |  |  | X |  |  | X |  | X |  |  |  |  |  |  | X |  |  |  |
| V_GTG |  | X | X |  |  |  |  | X | X |  | X |  |  | X | X |  |  |  |  | X | X |  |  |  |  | X | X |  |  |  | X | X | X |  | X |  |  | X | X |  | X |
| V_GTT | X |  | X | X | X |  |  |  | X |  | X |  | X |  | X | X |  |  |  |  | X | X | X |  | X |  | X | X | X |  |  |  | X | X | X |  | X |  | X | X | X |
| Y_TAC |  | X | X | X | X |  |  |  | X | X | X |  |  |  | X |  |  |  | X | X | X |  | X |  |  |  | X | X | X |  | X | X | X |  |  |  |  | X | X |  |  |
| Y_TAT |  |  |  |  |  |  |  |  |  |  |  |  | X |  |  |  |  |  |  |  |  |  |  |  | X |  |  |  |  |  |  |  |  | X |  |  | X |  |  | X |  |
| K_AAA |  |  |  |  |  |  |  |  |  |  | X |  |  |  |  |  |  |  |  |  |  |  |  |  | X |  |  |  |  |  |  |  |  |  |  |  | X |  |  |  |  |
| K_AAG |  | X | X | X | X |  | X | X | X | X |  |  | X | X | X | X | X |  | X | X | X | X | X |  |  | X | X | X | X |  | X | X | X | X | X |  |  | X | X | X | X |
