# Supplementary material for: The Evolutionary Basis of Translational Accuracy in Plants
Source: G3 (Bethesda). 2017 May 22;7(7):2363–73. doi: 10.1534/g3.117.040626 (PMC5499143; doi:10.1534/g3.117.040626)
Supplement: Supplementary file 6 [file 2363TableS6.docx]

**Table S6:** Correlations between the codon odds ratio calculated in domain vs non-domain regions, and in stem vs loop regions. Parametric and non-parametric statistics were calculated and the reported values are all significant (p < 0.0001).

|  | **Correlaiton odds ratio Domain/Non-domain** | | **Correlaiton odds ratio Stem/loop** | |
| --- | --- | --- | --- | --- |
| **Species** | **pearson r** | **spearman p** | **pearson r** | **spearman p** |
| AT | 0.92 | 0.93 | 0.9 | 0.93 |
| GM | 0.54 | 0.64 | 0.3 | 0.29 |
| MT | 0.89 | 0.86 | 0.86 | 0.85 |
| OS(HGC) | 0.86 | 0.85 | 0.95 | 0.95 |
| OS(LGC) | 0.87 | 0.87 | 0.87 | 0.9 |
| ZM(HGC) | 0.89 | 0.91 | 0.93 | 0.96 |
| ZM(LGC) | 0.92 | 0.92 | 0.73 | 0.82 |
