# Supplementary material for: The Evolutionary Basis of Translational Accuracy in Plants
Source: G3 (Bethesda). 2017 May 22;7(7):2363–73. doi: 10.1534/g3.117.040626 (PMC5499143; doi:10.1534/g3.117.040626)
Supplement: Supplementary file 7 [file 2363TableS7.docx]

**Table S7 a:** Odds ratios for codon enrichment analysis within the transcript portions representing protein domains. For each value, the corresponding significance is also reported.

| **Codon** | **AT** | **AT_sign** | **MT** | **MT_sign** | **OS(LGC)** | **OS(LGC)_sign** | **ZM(LGC)** | **ZM(LGC)_sign** | **OS(HGC)** | **OS(HGC)_sign** | **ZM(HGC)** | **ZM(HGC)_sign** |
| --- | --- | --- | --- | --- | --- | --- | --- | --- | --- | --- | --- | --- |
| **A_GCA** | 0.99 | 0.21 | 1.05 | 1.61 | 1.06 | 2.16 | 1.10 | 2.91 | 1.20 | 2.84 | 1.03 | 0.51 |
| **A_GCC** | 0.97 | 0.61 | 1.03 | 0.77 | 0.89 | 4.32 | 0.87 | 4.34 | 0.92 | 3.98 | 0.96 | 1.46 |
| **A_GCG** | 0.76 | 6.10 | 0.64 | 10.04 | 0.85 | 5.14 | 0.87 | 3.78 | 1.06 | 2.45 | 1.05 | 1.78 |
| **A_GCT** | 1.19 | 5.00 | 1.09 | 3.14 | 1.15 | 5.54 | 1.13 | 3.99 | 1.08 | 1.26 | 0.93 | 1.19 |
| **C_TGC** | 1.55 | 4.52 | 1.36 | 4.56 | 1.34 | 4.88 | 1.51 | 5.78 | 1.75 | 3.12 | 1.38 | 1.79 |
| **C_TGT** | 0.65 | 4.52 | 0.74 | 4.56 | 0.75 | 4.88 | 0.66 | 5.78 | 0.57 | 3.12 | 0.72 | 1.79 |
| **D_GAC** | 0.90 | 2.71 | 0.94 | 1.74 | 0.93 | 2.66 | 0.87 | 3.82 | 1.30 | 4.80 | 1.59 | 7.60 |
| **D_GAT** | 1.11 | 2.71 | 1.06 | 1.74 | 1.08 | 2.66 | 1.15 | 3.82 | 0.77 | 4.80 | 0.63 | 7.60 |
| **E_GAA** | 0.86 | 4.26 | 0.83 | 6.53 | 0.92 | 3.07 | 0.85 | 4.85 | 0.69 | 6.04 | 0.59 | 7.95 |
| **E_GAG** | 1.16 | 4.26 | 1.20 | 6.53 | 1.09 | 3.07 | 1.18 | 4.85 | 1.45 | 6.04 | 1.70 | 7.95 |
| **F_TTC** | 1.41 | 6.65 | 1.19 | 4.46 | 1.15 | 3.44 | 1.28 | 4.78 | 1.15 | 1.12 | 1.57 | 3.82 |
| **F_TTT** | 0.71 | 6.65 | 0.84 | 4.46 | 0.87 | 3.44 | 0.78 | 4.78 | 0.87 | 1.12 | 0.64 | 3.82 |
| **G_GGA** | 1.54 | 8.58 | 1.42 | 9.37 | 1.40 | 8.92 | 1.39 | 6.96 | 1.10 | 1.26 | 1.14 | 1.78 |
| **G_GGC** | 0.79 | 3.74 | 0.82 | 4.07 | 0.77 | 7.44 | 0.75 | 7.07 | 0.85 | 5.13 | 0.90 | 2.96 |
| **G_GGG** | 1.04 | 0.56 | 1.33 | 5.63 | 1.11 | 2.76 | 1.31 | 5.45 | 1.21 | 5.46 | 1.15 | 3.54 |
| **G_GGT** | 0.73 | 6.58 | 0.70 | 10.62 | 0.87 | 4.15 | 0.81 | 4.91 | 0.92 | 1.21 | 0.82 | 2.59 |
| **H_CAC** | 0.94 | 1.12 | 1.01 | 0.10 | 1.07 | 1.54 | 0.96 | 0.76 | 1.48 | 4.73 | 1.31 | 2.68 |
| **H_CAT** | 1.07 | 1.12 | 0.99 | 0.10 | 0.94 | 1.54 | 1.04 | 0.76 | 0.68 | 4.73 | 0.76 | 2.68 |
| **I_ATA** | 0.73 | 8.79 | 0.98 | 0.88 | 0.94 | 2.19 | 0.97 | 0.70 | 0.95 | 0.68 | 0.87 | 1.54 |
| **I_ATC** | 1.45 | 10.33 | 1.01 | 0.41 | 1.04 | 1.60 | 1.03 | 0.81 | 1.04 | 0.75 | 1.15 | 2.01 |
| **I_ATT** | 0.93 | 2.12 | 1.01 | 0.43 | 1.01 | 0.44 | 1.00 | 0.11 | 0.98 | 0.27 | 0.90 | 1.03 |
| **L_CTA** | 1.06 | 1.05 | 1.05 | 1.10 | 1.01 | 0.14 | 1.08 | 1.57 | 0.83 | 1.57 | 0.92 | 0.76 |
| **L_CTC** | 1.11 | 2.30 | 1.03 | 0.64 | 1.00 | 0.01 | 1.14 | 3.33 | 1.04 | 1.30 | 1.27 | 7.24 |
| **L_CTG** | 0.94 | 1.00 | 1.06 | 1.28 | 1.16 | 4.71 | 1.06 | 1.47 | 1.20 | 5.38 | 1.04 | 1.26 |
| **L_CTT** | 1.13 | 2.99 | 1.19 | 6.34 | 1.13 | 4.21 | 1.16 | 4.06 | 1.09 | 0.96 | 0.97 | 0.30 |
| **L_TTA** | 0.75 | 6.59 | 0.77 | 8.17 | 0.79 | 5.97 | 0.79 | 4.72 | 0.50 | 3.89 | 0.32 | 6.69 |
| **L_TTG** | 1.01 | 0.27 | 0.95 | 1.76 | 0.86 | 5.02 | 0.77 | 7.16 | 0.46 | 13.14 | 0.35 | 17.30 |
| **N_AAC** | 1.29 | 6.97 | 1.07 | 2.29 | 1.15 | 4.74 | 1.12 | 2.85 | 1.39 | 4.62 | 1.67 | 5.42 |
| **N_AAT** | 0.77 | 6.97 | 0.94 | 2.29 | 0.87 | 4.74 | 0.89 | 2.85 | 0.72 | 4.62 | 0.60 | 5.42 |
| **P_CCA** | 1.10 | 1.52 | 0.96 | 0.94 | 1.02 | 0.38 | 1.10 | 1.77 | 1.17 | 1.71 | 1.04 | 0.37 |
| **P_CCC** | 0.76 | 3.09 | 0.82 | 2.91 | 0.88 | 2.36 | 0.85 | 2.67 | 0.87 | 2.89 | 0.81 | 4.45 |
| **P_CCG** | 1.00 | 0.03 | 0.79 | 3.37 | 0.84 | 3.12 | 0.85 | 2.62 | 1.10 | 2.23 | 1.17 | 3.42 |
| **P_CCT** | 1.04 | 0.56 | 1.24 | 4.82 | 1.18 | 3.77 | 1.14 | 2.46 | 0.93 | 0.80 | 1.11 | 1.10 |
| **Q_CAA** | 0.86 | 3.16 | 0.75 | 7.63 | 0.83 | 5.52 | 0.80 | 5.00 | 0.66 | 5.07 | 0.63 | 5.13 |
| **Q_CAG** | 1.17 | 3.16 | 1.33 | 7.63 | 1.21 | 5.52 | 1.25 | 5.00 | 1.52 | 5.07 | 1.59 | 5.13 |
| **R_AGA** | 0.91 | 2.23 | 0.95 | 1.48 | 0.81 | 6.41 | 0.80 | 5.53 | 0.64 | 5.18 | 0.68 | 4.53 |
| **R_AGG** | 0.78 | 5.15 | 0.98 | 0.60 | 0.85 | 5.24 | 0.82 | 5.47 | 0.54 | 17.53 | 0.50 | 18.84 |
| **R_CGA** | 1.39 | 4.73 | 1.10 | 1.73 | 1.12 | 2.25 | 1.09 | 1.32 | 1.18 | 1.41 | 0.92 | 0.74 |
| **R_CGC** | 0.99 | 0.12 | 1.12 | 1.75 | 1.30 | 6.15 | 1.43 | 6.85 | 1.47 | 11.20 | 1.56 | 12.32 |
| **R_CGG** | 1.31 | 3.53 | 1.09 | 1.36 | 1.19 | 3.82 | 1.18 | 3.13 | 1.25 | 6.04 | 1.23 | 5.51 |
| **R_CGT** | 1.10 | 1.62 | 0.95 | 1.10 | 1.14 | 2.93 | 1.16 | 2.72 | 0.93 | 0.70 | 1.19 | 1.72 |
| **S_AGC** | 0.80 | 5.85 | 0.89 | 3.33 | 0.77 | 9.56 | 0.65 | 13.36 | 0.75 | 9.38 | 0.57 | 17.35 |
| **S_AGT** | 0.51 | 21.11 | 0.67 | 16.21 | 0.65 | 15.76 | 0.60 | 14.70 | 0.66 | 4.21 | 0.39 | 10.00 |
| **S_TCA** | 1.37 | 8.37 | 1.26 | 9.10 | 1.38 | 12.09 | 1.49 | 11.59 | 1.20 | 2.29 | 1.46 | 3.92 |
| **S_TCC** | 1.24 | 4.69 | 1.03 | 0.77 | 1.11 | 3.62 | 1.34 | 7.60 | 1.17 | 5.28 | 1.52 | 12.22 |
| **S_TCG** | 1.21 | 3.96 | 1.02 | 0.38 | 1.15 | 3.54 | 1.09 | 1.94 | 1.15 | 4.57 | 1.31 | 7.33 |
| **S_TCT** | 1.33 | 8.56 | 1.19 | 7.05 | 1.13 | 4.55 | 1.20 | 5.37 | 0.94 | 0.68 | 0.95 | 0.54 |
| **T_ACA** | 1.28 | 6.59 | 1.20 | 6.58 | 1.24 | 7.36 | 1.20 | 5.05 | 0.96 | 0.46 | 1.05 | 0.47 |
| **T_ACC** | 0.92 | 2.02 | 0.87 | 4.28 | 0.88 | 4.03 | 0.94 | 1.43 | 0.88 | 3.78 | 0.89 | 3.01 |
| **T_ACG** | 0.82 | 4.38 | 0.72 | 7.19 | 0.87 | 3.52 | 0.85 | 3.38 | 1.17 | 4.35 | 1.15 | 3.60 |
| **T_ACT** | 0.95 | 1.46 | 1.03 | 0.91 | 0.96 | 1.42 | 0.95 | 1.41 | 0.93 | 0.75 | 0.85 | 1.87 |
| **V_GTA** | 0.59 | 13.87 | 0.69 | 12.29 | 0.82 | 6.10 | 0.69 | 9.81 | 0.78 | 2.31 | 0.63 | 5.02 |
| **V_GTC** | 0.90 | 2.75 | 0.96 | 1.30 | 0.93 | 2.69 | 0.93 | 2.22 | 0.77 | 9.39 | 0.61 | 16.36 |
| **V_GTG** | 1.49 | 10.47 | 1.44 | 12.25 | 1.26 | 8.92 | 1.36 | 9.90 | 1.32 | 10.08 | 1.75 | 18.38 |
| **V_GTT** | 1.10 | 2.92 | 0.99 | 0.46 | 0.96 | 1.72 | 0.98 | 0.56 | 0.97 | 0.45 | 0.88 | 1.70 |
| **Y_TAC** | 1.46 | 5.90 | 1.25 | 4.73 | 1.20 | 3.70 | 1.30 | 4.34 | 1.48 | 3.15 | 1.40 | 2.30 |
| **Y_TAT** | 0.68 | 5.90 | 0.80 | 4.73 | 0.84 | 3.70 | 0.77 | 4.34 | 0.68 | 3.15 | 0.71 | 2.30 |
| **K_AAA** | 0.77 | 7.33 | 0.83 | 7.09 | 0.80 | 8.04 | 0.73 | 8.76 | 0.77 | 3.47 | 0.82 | 2.20 |
| **K_AAG** | 1.29 | 7.33 | 1.20 | 7.09 | 1.25 | 8.04 | 1.37 | 8.76 | 1.30 | 3.47 | 1.23 | 2.20 |

**Table S7 b:** Odds ratios for codon enrichment analysis within the transcript portions representing non-domains. For each value, the corresponding significance is also reported.

| **Codon** | **AT** | **AT_sign** | **MT** | **MT_sign** | **OS(LGC)** | **OS(LGC)_sign** | **ZM(LGC)** | **ZM(LGC)_sign** | **OS(HGC)** | **OS(HGC)_sign** | **ZM(HGC)** | **ZM(HGC)_sign** |
| --- | --- | --- | --- | --- | --- | --- | --- | --- | --- | --- | --- | --- |
| **A_GCA** | 0.99 | 0.26 | 1.01 | 0.49 | 1.09 | 4.36 | 1.02 | 0.64 | 0.93 | 1.64 | 0.85 | 3.46 |
| **A_GCC** | 0.85 | 4.57 | 0.96 | 1.29 | 0.94 | 3.04 | 0.93 | 2.93 | 0.98 | 0.80 | 0.97 | 1.38 |
| **A_GCG** | 0.84 | 4.52 | 0.68 | 9.54 | 0.86 | 7.72 | 0.98 | 0.86 | 1.08 | 3.49 | 1.15 | 5.46 |
| **A_GCT** | 1.20 | 6.63 | 1.14 | 5.58 | 1.12 | 5.84 | 1.07 | 2.95 | 0.86 | 3.68 | 0.85 | 3.70 |
| **C_TGC** | 1.31 | 3.72 | 1.33 | 4.99 | 1.46 | 7.61 | 1.40 | 5.95 | 1.72 | 3.95 | 1.78 | 3.90 |
| **C_TGT** | 0.77 | 3.72 | 0.75 | 4.99 | 0.68 | 7.61 | 0.72 | 5.95 | 0.58 | 3.95 | 0.56 | 3.90 |
| **D_GAC** | 0.77 | 8.21 | 0.88 | 4.55 | 0.94 | 2.80 | 0.88 | 4.59 | 1.32 | 5.54 | 1.28 | 4.14 |
| **D_GAT** | 1.30 | 8.21 | 1.14 | 4.55 | 1.07 | 2.80 | 1.14 | 4.59 | 0.76 | 5.54 | 0.78 | 4.14 |
| **E_GAA** | 0.87 | 4.90 | 0.87 | 5.84 | 0.99 | 0.28 | 0.91 | 3.76 | 0.68 | 6.85 | 0.57 | 9.18 |
| **E_GAG** | 1.15 | 4.90 | 1.15 | 5.84 | 1.01 | 0.28 | 1.10 | 3.76 | 1.48 | 6.85 | 1.76 | 9.19 |
| **F_TTC** | 1.30 | 6.48 | 1.05 | 1.45 | 1.18 | 4.56 | 1.11 | 2.44 | 1.50 | 3.84 | 1.40 | 3.26 |
| **F_TTT** | 0.77 | 6.48 | 0.95 | 1.45 | 0.85 | 4.57 | 0.90 | 2.44 | 0.66 | 3.84 | 0.71 | 3.26 |
| **G_GGA** | 1.55 | 12.14 | 1.48 | 13.29 | 1.33 | 11.36 | 1.23 | 6.72 | 1.03 | 0.61 | 1.03 | 0.43 |
| **G_GGC** | 0.82 | 4.38 | 0.87 | 3.67 | 0.80 | 10.08 | 0.85 | 5.86 | 0.92 | 2.92 | 0.96 | 1.19 |
| **G_GGG** | 1.12 | 2.53 | 1.27 | 6.45 | 1.16 | 5.89 | 1.22 | 6.33 | 1.28 | 6.97 | 1.23 | 5.10 |
| **G_GGT** | 0.69 | 10.99 | 0.66 | 15.32 | 0.86 | 6.58 | 0.83 | 6.51 | 0.73 | 6.19 | 0.70 | 6.13 |
| **H_CAC** | 1.03 | 0.64 | 0.97 | 0.81 | 1.03 | 0.88 | 1.00 | 0.06 | 1.29 | 3.40 | 1.58 | 5.04 |
| **H_CAT** | 0.97 | 0.64 | 1.03 | 0.81 | 0.97 | 0.88 | 1.00 | 0.06 | 0.78 | 3.40 | 0.63 | 5.04 |
| **I_ATA** | 0.74 | 9.49 | 0.91 | 3.35 | 0.94 | 2.28 | 0.93 | 2.20 | 0.96 | 0.36 | 0.98 | 0.08 |
| **I_ATC** | 1.44 | 11.04 | 1.06 | 2.00 | 1.09 | 3.22 | 1.10 | 2.97 | 1.28 | 3.49 | 1.29 | 3.17 |
| **I_ATT** | 0.94 | 1.93 | 1.03 | 1.31 | 0.98 | 0.96 | 0.97 | 0.80 | 0.72 | 3.86 | 0.68 | 3.87 |
| **L_CTA** | 0.98 | 0.51 | 1.02 | 0.51 | 1.06 | 1.75 | 1.05 | 1.28 | 0.75 | 2.94 | 0.80 | 2.28 |
| **L_CTC** | 1.21 | 4.81 | 1.00 | 0.11 | 1.03 | 1.18 | 1.09 | 2.93 | 1.19 | 5.04 | 1.29 | 7.17 |
| **L_CTG** | 1.00 | 0.04 | 1.16 | 3.66 | 1.08 | 2.90 | 1.03 | 1.03 | 1.19 | 4.91 | 1.11 | 2.94 |
| **L_CTT** | 1.07 | 2.07 | 1.15 | 5.65 | 1.08 | 3.22 | 1.19 | 5.75 | 0.92 | 1.14 | 0.84 | 2.51 |
| **L_TTA** | 0.79 | 6.76 | 0.74 | 11.09 | 0.85 | 4.90 | 0.81 | 5.51 | 0.51 | 4.47 | 0.60 | 3.08 |
| **L_TTG** | 1.00 | 0.01 | 1.03 | 1.27 | 0.88 | 5.09 | 0.82 | 6.94 | 0.49 | 13.12 | 0.42 | 14.44 |
| **N_AAC** | 1.25 | 7.42 | 0.94 | 2.61 | 1.09 | 3.17 | 1.05 | 1.64 | 1.69 | 6.64 | 1.71 | 5.56 |
| **N_AAT** | 0.80 | 7.42 | 1.07 | 2.61 | 0.92 | 3.17 | 0.95 | 1.64 | 0.59 | 6.64 | 0.59 | 5.56 |
| **P_CCA** | 1.05 | 1.39 | 0.97 | 0.97 | 1.05 | 1.96 | 1.10 | 3.12 | 0.84 | 3.08 | 0.92 | 1.44 |
| **P_CCC** | 0.79 | 4.37 | 0.81 | 4.99 | 0.88 | 4.68 | 0.84 | 4.96 | 0.99 | 0.16 | 0.92 | 1.87 |
| **P_CCG** | 0.95 | 1.06 | 0.87 | 3.23 | 0.90 | 3.63 | 0.88 | 3.70 | 1.12 | 3.25 | 1.18 | 4.27 |
| **P_CCT** | 1.09 | 2.26 | 1.20 | 6.36 | 1.14 | 5.19 | 1.13 | 4.17 | 0.90 | 1.84 | 0.85 | 2.63 |
| **Q_CAA** | 0.92 | 2.18 | 0.79 | 7.65 | 0.94 | 2.13 | 0.91 | 2.99 | 0.80 | 3.07 | 0.66 | 5.02 |
| **Q_CAG** | 1.09 | 2.18 | 1.27 | 7.65 | 1.06 | 2.13 | 1.10 | 2.99 | 1.25 | 3.07 | 1.51 | 5.02 |
| **R_AGA** | 1.04 | 1.15 | 1.13 | 4.13 | 0.95 | 1.83 | 0.89 | 3.62 | 0.61 | 6.71 | 0.70 | 4.67 |
| **R_AGG** | 0.93 | 1.90 | 1.09 | 2.71 | 0.93 | 2.91 | 1.00 | 0.13 | 0.70 | 9.10 | 0.69 | 8.91 |
| **R_CGA** | 1.23 | 3.73 | 0.96 | 0.79 | 1.06 | 1.27 | 1.03 | 0.54 | 0.70 | 4.13 | 0.79 | 2.53 |
| **R_CGC** | 0.91 | 1.34 | 0.98 | 0.45 | 1.20 | 5.86 | 1.19 | 4.71 | 1.28 | 6.21 | 1.33 | 6.96 |
| **R_CGG** | 1.14 | 2.15 | 0.95 | 0.91 | 1.13 | 3.77 | 1.12 | 2.88 | 1.47 | 9.33 | 1.30 | 6.00 |
| **R_CGT** | 0.85 | 3.63 | 0.77 | 7.05 | 0.80 | 6.04 | 0.83 | 4.34 | 0.75 | 3.35 | 0.83 | 2.13 |
| **S_AGC** | 0.75 | 9.69 | 0.81 | 7.53 | 0.73 | 14.92 | 0.68 | 15.47 | 0.82 | 6.32 | 0.71 | 10.41 |
| **S_AGT** | 0.49 | 28.99 | 0.62 | 22.65 | 0.60 | 23.47 | 0.56 | 22.64 | 0.54 | 7.86 | 0.48 | 8.62 |
| **S_TCA** | 1.36 | 11.17 | 1.23 | 10.68 | 1.36 | 15.40 | 1.42 | 14.35 | 0.99 | 0.18 | 1.00 | 0.02 |
| **S_TCC** | 1.26 | 6.56 | 1.07 | 2.53 | 1.15 | 6.62 | 1.31 | 10.30 | 1.18 | 5.51 | 1.28 | 7.31 |
| **S_TCG** | 1.09 | 2.47 | 0.94 | 1.85 | 0.99 | 0.58 | 1.07 | 1.97 | 1.14 | 4.40 | 1.27 | 6.80 |
| **S_TCT** | 1.41 | 14.01 | 1.27 | 12.46 | 1.29 | 12.62 | 1.23 | 8.56 | 0.92 | 1.31 | 0.97 | 0.40 |
| **T_ACA** | 1.36 | 10.53 | 1.22 | 8.07 | 1.20 | 7.35 | 1.26 | 8.00 | 0.89 | 1.58 | 0.86 | 1.91 |
| **T_ACC** | 0.88 | 3.85 | 0.83 | 6.33 | 0.92 | 3.16 | 0.86 | 4.80 | 0.89 | 3.04 | 0.99 | 0.31 |
| **T_ACG** | 0.92 | 2.44 | 0.73 | 7.60 | 0.87 | 4.30 | 0.81 | 5.42 | 1.20 | 4.48 | 1.15 | 3.16 |
| **T_ACT** | 0.87 | 5.21 | 1.04 | 1.49 | 0.97 | 1.38 | 1.01 | 0.43 | 0.91 | 1.10 | 0.77 | 3.19 |
| **V_GTA** | 0.69 | 11.59 | 0.79 | 8.15 | 0.84 | 6.16 | 0.84 | 5.16 | 0.65 | 4.48 | 0.56 | 6.25 |
| **V_GTC** | 0.95 | 1.38 | 0.95 | 1.38 | 0.99 | 0.26 | 0.92 | 3.02 | 0.92 | 2.52 | 0.76 | 7.47 |
| **V_GTG** | 1.17 | 5.09 | 1.11 | 3.93 | 1.02 | 0.98 | 1.13 | 4.61 | 1.21 | 5.93 | 1.56 | 12.28 |
| **V_GTT** | 1.16 | 5.49 | 1.09 | 3.74 | 1.09 | 3.93 | 1.05 | 2.04 | 0.78 | 3.81 | 0.71 | 4.89 |
| **Y_TAC** | 1.52 | 7.28 | 1.10 | 2.07 | 1.31 | 6.10 | 1.32 | 5.35 | 1.37 | 2.16 | 1.58 | 2.97 |
| **Y_TAT** | 0.66 | 7.28 | 0.91 | 2.07 | 0.77 | 6.10 | 0.76 | 5.35 | 0.73 | 2.16 | 0.63 | 2.97 |
| **K_AAA** | 0.83 | 6.34 | 0.84 | 7.43 | 0.85 | 6.87 | 0.77 | 8.93 | 0.73 | 3.95 | 0.69 | 4.45 |
| **K_AAG** | 1.21 | 6.34 | 1.19 | 7.43 | 1.18 | 6.88 | 1.31 | 8.93 | 1.37 | 3.95 | 1.45 | 4.45 |

**Table S7 c:** Odds ratios for codon enrichment analysis relative to the Mantel Heisenzl test between conserved sites in domains and conserved sites in non domains. For each value, the corresponding significance is also reported.

| **Codon** | **AT** | **AT_sign** | **MT** | **MT_sign** | **OS(LGC)** | **OS(LGC)_sign** | **ZM(LGC)** | **ZM(LGC)_sign** | **OS(HGC)** | **OS(HGC)_sign** | **ZM(HGC)** | **ZM(HGC)_sign** |
| --- | --- | --- | --- | --- | --- | --- | --- | --- | --- | --- | --- | --- |
| **A_GCA** | 1.09 | 7.07 | 1.02 | 1.26 | 1.53 | 26.81 | 1.31 | 13.86 | 0.87 | 3.37 | 0.71 | 7.78 |
| **A_GCC** | 0.89 | 7.45 | 0.91 | 3.90 | 0.73 | 19.31 | 0.74 | 14.83 | 1.24 | 11.43 | 1.20 | 8.57 |
| **A_GCG** | 0.91 | 6.15 | 0.93 | 2.27 | 0.44 | 45.26 | 0.57 | 24.64 | 0.86 | 7.81 | 0.98 | 0.84 |
| **A_GCT** | 1.03 | 3.07 | 1.05 | 2.63 | 1.54 | 28.13 | 1.44 | 19.50 | 0.83 | 4.80 | 0.71 | 8.31 |
| **C_TGC** | 1.01 | 0.37 | 1.03 | 0.77 | 0.83 | 5.61 | 0.91 | 2.23 | 1.61 | 4.78 | 1.66 | 4.63 |
| **C_TGT** | 0.99 | 0.37 | 0.97 | 0.77 | 1.20 | 5.61 | 1.09 | 2.23 | 0.62 | 4.78 | 0.60 | 4.63 |
| **D_GAC** | 1.05 | 3.54 | 1.00 | 0.13 | 0.85 | 9.62 | 0.90 | 4.63 | 1.33 | 6.78 | 1.48 | 7.50 |
| **D_GAT** | 0.95 | 3.54 | 1.00 | 0.13 | 1.18 | 9.62 | 1.11 | 4.63 | 0.75 | 6.78 | 0.68 | 7.50 |
| **E_GAA** | 0.91 | 8.63 | 0.88 | 7.47 | 1.14 | 8.05 | 1.05 | 2.69 | 0.82 | 3.71 | 0.72 | 5.77 |
| **E_GAG** | 1.10 | 8.63 | 1.13 | 7.47 | 0.88 | 8.05 | 0.95 | 2.69 | 1.22 | 3.71 | 1.40 | 5.77 |
| **F_TTC** | 0.89 | 7.94 | 0.85 | 7.40 | 0.77 | 12.14 | 0.82 | 7.51 | 1.04 | 0.58 | 1.53 | 5.72 |
| **F_TTT** | 1.12 | 7.94 | 1.17 | 7.40 | 1.29 | 12.14 | 1.22 | 7.51 | 0.96 | 0.58 | 0.65 | 5.72 |
| **G_GGA** | 1.00 | 0.39 | 0.99 | 0.76 | 1.21 | 10.98 | 1.20 | 8.33 | 0.69 | 8.64 | 0.70 | 7.72 |
| **G_GGC** | 0.93 | 4.48 | 1.02 | 0.76 | 0.77 | 15.57 | 0.77 | 12.33 | 1.12 | 4.81 | 1.15 | 5.33 |
| **G_GGG** | 1.09 | 5.66 | 1.01 | 0.47 | 0.84 | 9.91 | 0.92 | 3.82 | 1.06 | 2.46 | 1.09 | 2.85 |
| **G_GGT** | 0.98 | 1.63 | 1.00 | 0.15 | 1.27 | 13.59 | 1.18 | 7.52 | 0.79 | 5.16 | 0.68 | 7.51 |
| **H_CAC** | 0.99 | 0.34 | 0.92 | 2.46 | 0.83 | 6.58 | 0.84 | 5.02 | 1.36 | 4.60 | 1.34 | 3.47 |
| **H_CAT** | 1.01 | 0.34 | 1.08 | 2.46 | 1.21 | 6.58 | 1.19 | 5.02 | 0.73 | 4.60 | 0.74 | 3.47 |
| **I_ATA** | 1.12 | 7.46 | 1.11 | 4.64 | 1.10 | 4.20 | 1.10 | 3.51 | 0.89 | 1.45 | 0.87 | 1.60 |
| **I_ATC** | 0.88 | 9.76 | 0.86 | 6.35 | 0.82 | 9.27 | 0.83 | 7.37 | 1.18 | 2.89 | 1.26 | 3.64 |
| **I_ATT** | 1.04 | 2.73 | 1.02 | 1.02 | 1.11 | 4.99 | 1.10 | 3.86 | 0.85 | 2.26 | 0.77 | 3.19 |
| **L_CTA** | 1.03 | 1.91 | 0.96 | 1.63 | 1.19 | 7.89 | 1.06 | 2.21 | 0.94 | 0.88 | 0.79 | 3.48 |
| **L_CTC** | 0.88 | 10.34 | 0.76 | 13.56 | 0.66 | 27.90 | 0.75 | 15.17 | 1.17 | 7.83 | 1.01 | 0.55 |
| **L_CTG** | 1.03 | 1.74 | 1.12 | 4.83 | 0.85 | 10.29 | 0.89 | 6.15 | 0.95 | 2.41 | 1.12 | 5.23 |
| **L_CTT** | 1.11 | 10.39 | 1.13 | 8.21 | 1.34 | 19.44 | 1.24 | 11.45 | 0.84 | 4.05 | 0.81 | 4.52 |
| **L_TTA** | 0.92 | 6.48 | 0.93 | 3.92 | 1.14 | 5.81 | 1.11 | 3.57 | 0.81 | 1.75 | 0.64 | 3.03 |
| **L_TTG** | 1.01 | 0.79 | 1.04 | 2.33 | 1.13 | 7.65 | 1.11 | 5.15 | 0.74 | 7.21 | 0.73 | 5.60 |
| **N_AAC** | 1.05 | 3.74 | 0.96 | 2.03 | 0.93 | 3.32 | 0.93 | 2.64 | 1.27 | 3.51 | 1.52 | 5.11 |
| **N_AAT** | 0.95 | 3.74 | 1.05 | 2.03 | 1.08 | 3.32 | 1.08 | 2.64 | 0.79 | 3.51 | 0.66 | 5.11 |
| **P_CCA** | 1.04 | 2.62 | 1.00 | 0.24 | 1.43 | 19.36 | 1.25 | 9.92 | 0.95 | 1.09 | 0.77 | 5.39 |
| **P_CCC** | 0.95 | 2.51 | 0.91 | 3.27 | 0.77 | 11.21 | 0.82 | 6.98 | 1.37 | 10.64 | 1.33 | 8.97 |
| **P_CCG** | 0.89 | 6.50 | 0.96 | 1.25 | 0.49 | 32.07 | 0.60 | 18.58 | 0.83 | 7.18 | 0.93 | 2.51 |
| **P_CCT** | 1.05 | 4.02 | 1.06 | 3.09 | 1.34 | 15.58 | 1.27 | 10.36 | 0.85 | 3.45 | 0.77 | 4.99 |
| **Q_CAA** | 0.91 | 6.33 | 0.80 | 9.07 | 1.07 | 3.13 | 0.97 | 1.00 | 0.90 | 1.52 | 0.86 | 1.84 |
| **Q_CAG** | 1.10 | 6.33 | 1.24 | 9.07 | 0.93 | 3.13 | 1.03 | 1.00 | 1.11 | 1.52 | 1.17 | 1.84 |
| **R_AGA** | 0.93 | 5.87 | 0.88 | 6.31 | 1.20 | 9.53 | 1.14 | 5.39 | 0.82 | 3.26 | 0.69 | 5.37 |
| **R_AGG** | 1.07 | 4.65 | 1.01 | 0.32 | 0.97 | 1.85 | 1.00 | 0.15 | 0.79 | 8.16 | 0.80 | 6.50 |
| **R_CGA** | 0.96 | 1.99 | 1.10 | 3.37 | 1.26 | 8.06 | 1.15 | 3.93 | 0.85 | 2.36 | 0.73 | 4.07 |
| **R_CGC** | 0.99 | 0.38 | 0.91 | 2.82 | 0.67 | 19.28 | 0.72 | 12.72 | 1.43 | 14.68 | 1.34 | 10.54 |
| **R_CGG** | 1.02 | 1.07 | 1.12 | 3.32 | 0.77 | 11.66 | 0.85 | 5.69 | 0.87 | 5.50 | 1.00 | 0.10 |
| **R_CGT** | 1.06 | 3.82 | 1.12 | 4.57 | 1.63 | 18.54 | 1.41 | 10.82 | 0.93 | 1.09 | 0.76 | 3.99 |
| **S_AGC** | 1.22 | 14.56 | 1.18 | 6.72 | 1.06 | 3.14 | 1.02 | 0.93 | 1.19 | 6.12 | 1.11 | 3.50 |
| **S_AGT** | 1.22 | 15.57 | 1.33 | 14.71 | 1.36 | 14.44 | 1.29 | 9.34 | 0.90 | 1.13 | 0.86 | 1.52 |
| **S_TCA** | 0.98 | 2.23 | 0.91 | 5.76 | 1.18 | 9.87 | 1.13 | 5.91 | 0.91 | 1.57 | 0.77 | 3.96 |
| **S_TCC** | 0.86 | 10.95 | 0.81 | 9.82 | 0.70 | 19.62 | 0.75 | 12.49 | 1.16 | 5.70 | 1.15 | 4.71 |
| **S_TCG** | 1.00 | 0.12 | 1.09 | 2.85 | 0.64 | 19.05 | 0.76 | 9.73 | 0.80 | 8.15 | 0.89 | 3.67 |
| **S_TCT** | 0.87 | 13.29 | 0.92 | 5.45 | 1.13 | 6.85 | 1.09 | 3.86 | 0.69 | 5.97 | 0.71 | 5.38 |
| **T_ACA** | 1.09 | 6.28 | 1.01 | 0.57 | 1.18 | 7.77 | 1.15 | 5.33 | 1.01 | 0.17 | 0.81 | 2.93 |
| **T_ACC** | 0.90 | 6.38 | 0.82 | 7.27 | 0.81 | 8.72 | 0.82 | 6.72 | 1.16 | 4.30 | 1.12 | 2.97 |
| **T_ACG** | 0.91 | 5.20 | 1.10 | 2.30 | 0.70 | 12.08 | 0.77 | 7.15 | 0.89 | 3.45 | 1.03 | 0.87 |
| **T_ACT** | 1.04 | 2.90 | 1.09 | 3.88 | 1.21 | 8.54 | 1.17 | 5.74 | 0.84 | 2.29 | 0.66 | 5.35 |
| **V_GTA** | 0.91 | 5.82 | 0.94 | 2.65 | 1.08 | 3.15 | 0.98 | 0.70 | 0.71 | 4.02 | 0.74 | 3.24 |
| **V_GTC** | 0.98 | 1.12 | 1.00 | 0.06 | 0.92 | 4.24 | 0.95 | 2.07 | 1.20 | 7.14 | 1.04 | 1.52 |
| **V_GTG** | 1.04 | 3.42 | 1.03 | 1.68 | 0.83 | 10.42 | 0.92 | 4.10 | 0.90 | 4.14 | 1.04 | 1.52 |
| **V_GTT** | 1.02 | 2.07 | 1.01 | 0.44 | 1.22 | 11.61 | 1.15 | 6.39 | 0.80 | 4.10 | 0.74 | 4.84 |
| **Y_TAC** | 0.99 | 0.29 | 0.94 | 2.27 | 0.79 | 9.31 | 0.86 | 4.86 | 1.06 | 0.73 | 1.24 | 2.23 |
| **Y_TAT** | 1.01 | 0.29 | 1.07 | 2.27 | 1.27 | 9.31 | 1.17 | 4.86 | 0.94 | 0.73 | 0.80 | 2.23 |
| **K_AAA** | 1.00 | 0.08 | 0.90 | 6.27 | 1.11 | 5.73 | 1.03 | 1.47 | 0.81 | 3.14 | 0.68 | 5.53 |
| **K_AAG** | 1.00 | 0.08 | 1.11 | 6.27 | 0.90 | 5.73 | 0.97 | 1.47 | 1.23 | 3.14 | 1.47 | 5.53 |

**Table S7 d:** Odds ratios for codon enrichment analysis, restricted to codons with the third base in a stem region. For each value, the corresponding significance is also reported.

| **Codon** | **AT** | **AT_sign** | **MT** | **MT_sign** | **OS(LGC)** | **OS(LGC)_sign** | **ZM(LGC)** | **ZM(LGC)_sign** | **OS(HGC)** | **OS(HGC)_sign** | **ZM(HGC)** | **ZM(HGC)_sign** |
| --- | --- | --- | --- | --- | --- | --- | --- | --- | --- | --- | --- | --- |
| **A_GCA** | 0.98 | 0.21 | 1.05 | 0.81 | 1.25 | 6.75 | 1.13 | 2.35 | 0.93 | 1.20 | 0.85 | 2.03 |
| **A_GCC** | 0.88 | 1.81 | 0.99 | 0.09 | 0.87 | 4.68 | 0.94 | 1.34 | 0.97 | 1.58 | 0.99 | 0.34 |
| **A_GCG** | 0.82 | 2.98 | 0.75 | 3.74 | 0.69 | 11.87 | 0.79 | 4.85 | 1.07 | 3.04 | 1.08 | 2.66 |
| **A_GCT** | 1.23 | 3.78 | 1.08 | 1.49 | 1.32 | 9.10 | 1.18 | 3.57 | 0.89 | 2.43 | 0.82 | 3.27 |
| **C_TGC** | 1.67 | 3.35 | 1.37 | 2.08 | 1.10 | 1.17 | 1.84 | 5.26 | 1.91 | 3.78 | 1.70 | 2.50 |
| **C_TGT** | 0.60 | 3.35 | 0.73 | 2.08 | 0.91 | 1.17 | 0.54 | 5.26 | 0.52 | 3.78 | 0.59 | 2.50 |
| **D_GAC** | 0.91 | 1.36 | 1.02 | 0.21 | 0.94 | 1.52 | 0.78 | 3.94 | 1.46 | 6.38 | 1.53 | 4.65 |
| **D_GAT** | 1.10 | 1.36 | 0.98 | 0.21 | 1.06 | 1.52 | 1.28 | 3.94 | 0.68 | 6.38 | 0.65 | 4.65 |
| **E_GAA** | 0.87 | 2.26 | 0.84 | 3.06 | 1.00 | 0.08 | 0.98 | 0.30 | 0.79 | 2.84 | 0.54 | 4.78 |
| **E_GAG** | 1.15 | 2.26 | 1.19 | 3.06 | 1.00 | 0.08 | 1.02 | 0.30 | 1.27 | 2.84 | 1.84 | 4.78 |
| **F_TTC** | 1.34 | 3.47 | 0.99 | 0.09 | 1.08 | 1.21 | 1.09 | 0.88 | 1.50 | 3.22 | 1.50 | 2.25 |
| **F_TTT** | 0.75 | 3.47 | 1.01 | 0.09 | 0.93 | 1.21 | 0.92 | 0.88 | 0.67 | 3.22 | 0.66 | 2.25 |
| **G_GGA** | 1.72 | 7.34 | 1.51 | 6.31 | 1.33 | 6.52 | 1.29 | 3.56 | 0.95 | 0.72 | 0.91 | 1.01 |
| **G_GGC** | 0.80 | 2.58 | 0.90 | 1.34 | 0.78 | 6.99 | 0.79 | 4.16 | 0.91 | 3.28 | 0.98 | 0.38 |
| **G_GGG** | 1.14 | 1.43 | 1.27 | 3.04 | 1.06 | 1.47 | 1.16 | 2.29 | 1.25 | 7.00 | 1.20 | 3.77 |
| **G_GGT** | 0.65 | 6.48 | 0.66 | 7.31 | 0.99 | 0.22 | 0.93 | 1.09 | 0.74 | 5.53 | 0.66 | 5.18 |
| **H_CAC** | 1.08 | 0.62 | 0.83 | 1.81 | 0.94 | 0.99 | 0.96 | 0.39 | 1.37 | 3.18 | 1.81 | 3.67 |
| **H_CAT** | 0.93 | 0.62 | 1.20 | 1.81 | 1.07 | 0.99 | 1.04 | 0.39 | 0.73 | 3.18 | 0.55 | 3.67 |
| **I_ATA** | 0.69 | 4.70 | 0.98 | 0.26 | 1.04 | 0.67 | 1.02 | 0.23 | 1.13 | 0.70 | 0.90 | 0.31 |
| **I_ATC** | 1.50 | 5.95 | 0.96 | 0.59 | 0.97 | 0.73 | 0.97 | 0.45 | 1.07 | 0.78 | 1.41 | 2.26 |
| **I_ATT** | 0.87 | 2.15 | 1.05 | 0.76 | 1.01 | 0.18 | 1.02 | 0.23 | 0.87 | 1.38 | 0.64 | 2.42 |
| **L_CTA** | 1.02 | 0.10 | 1.08 | 0.74 | 1.02 | 0.20 | 1.01 | 0.02 | 0.73 | 1.80 | 0.87 | 0.67 |
| **L_CTC** | 1.01 | 0.17 | 0.83 | 2.59 | 0.95 | 1.36 | 1.02 | 0.25 | 1.11 | 3.00 | 1.28 | 5.27 |
| **L_CTG** | 1.03 | 0.31 | 1.06 | 0.59 | 1.02 | 0.41 | 1.02 | 0.39 | 1.17 | 4.64 | 1.06 | 1.29 |
| **L_CTT** | 1.21 | 2.78 | 1.08 | 1.32 | 1.24 | 4.89 | 1.27 | 3.51 | 1.08 | 0.82 | 0.94 | 0.47 |
| **L_TTA** | 0.74 | 3.36 | 0.73 | 4.24 | 0.88 | 1.81 | 0.93 | 0.55 | 0.40 | 3.91 | 0.32 | 2.86 |
| **L_TTG** | 0.94 | 0.94 | 1.15 | 2.60 | 0.89 | 2.97 | 0.79 | 3.83 | 0.45 | 13.92 | 0.36 | 12.85 |
| **N_AAC** | 1.43 | 4.92 | 0.95 | 0.82 | 1.09 | 1.73 | 1.21 | 2.32 | 1.63 | 4.67 | 2.65 | 5.13 |
| **N_AAT** | 0.70 | 4.92 | 1.06 | 0.82 | 0.92 | 1.73 | 0.83 | 2.32 | 0.61 | 4.67 | 0.38 | 5.13 |
| **P_CCA** | 1.17 | 1.71 | 1.00 | 0.01 | 1.28 | 5.07 | 1.32 | 3.50 | 0.96 | 0.44 | 1.02 | 0.09 |
| **P_CCC** | 0.73 | 2.42 | 0.62 | 4.64 | 0.83 | 3.39 | 0.91 | 1.06 | 0.94 | 1.36 | 0.89 | 1.94 |
| **P_CCG** | 0.97 | 0.27 | 0.85 | 1.52 | 0.72 | 6.95 | 0.59 | 7.04 | 1.10 | 2.35 | 1.12 | 2.17 |
| **P_CCT** | 1.01 | 0.12 | 1.37 | 4.18 | 1.21 | 4.13 | 1.37 | 4.12 | 0.89 | 1.61 | 0.91 | 0.86 |
| **Q_CAA** | 1.03 | 0.32 | 0.71 | 4.28 | 0.89 | 2.05 | 0.91 | 0.99 | 0.59 | 4.06 | 0.56 | 2.80 |
| **Q_CAG** | 0.97 | 0.32 | 1.40 | 4.28 | 1.12 | 2.05 | 1.10 | 0.99 | 1.69 | 4.06 | 1.78 | 2.80 |
| **R_AGA** | 0.90 | 1.48 | 0.99 | 0.13 | 0.95 | 0.96 | 0.81 | 2.75 | 0.63 | 4.67 | 0.58 | 4.48 |
| **R_AGG** | 0.83 | 2.38 | 0.99 | 0.12 | 0.86 | 4.11 | 0.97 | 0.45 | 0.59 | 14.64 | 0.58 | 11.29 |
| **R_CGA** | 1.42 | 2.78 | 0.94 | 0.52 | 1.04 | 0.48 | 1.27 | 1.83 | 0.84 | 1.51 | 0.85 | 0.93 |
| **R_CGC** | 0.80 | 1.74 | 1.04 | 0.31 | 1.16 | 3.03 | 1.08 | 1.02 | 1.43 | 10.13 | 1.54 | 9.13 |
| **R_CGG** | 1.31 | 2.21 | 1.01 | 0.01 | 1.13 | 2.39 | 1.06 | 0.69 | 1.23 | 5.57 | 1.22 | 4.08 |
| **R_CGT** | 1.17 | 1.61 | 1.04 | 0.47 | 1.02 | 0.32 | 1.04 | 0.34 | 0.95 | 0.55 | 0.65 | 3.83 |
| **S_AGC** | 0.79 | 4.08 | 0.93 | 1.16 | 0.80 | 6.96 | 0.71 | 6.62 | 0.85 | 5.45 | 0.67 | 9.65 |
| **S_AGT** | 0.53 | 12.60 | 0.69 | 7.79 | 0.63 | 12.82 | 0.63 | 8.16 | 0.67 | 4.49 | 0.43 | 6.25 |
| **S_TCA** | 1.44 | 5.73 | 1.27 | 4.75 | 1.43 | 9.69 | 1.45 | 6.16 | 1.08 | 0.75 | 1.29 | 1.76 |
| **S_TCC** | 1.20 | 2.64 | 1.01 | 0.13 | 1.08 | 2.28 | 1.22 | 3.58 | 1.12 | 3.70 | 1.36 | 6.80 |
| **S_TCG** | 1.21 | 2.67 | 0.97 | 0.32 | 0.96 | 0.99 | 0.99 | 0.11 | 1.11 | 3.63 | 1.24 | 4.87 |
| **S_TCT** | 1.28 | 5.05 | 1.16 | 3.44 | 1.30 | 7.75 | 1.28 | 4.68 | 0.86 | 1.95 | 0.90 | 0.92 |
| **T_ACA** | 1.40 | 4.76 | 1.20 | 2.99 | 1.19 | 3.88 | 1.40 | 4.50 | 1.01 | 0.06 | 1.04 | 0.22 |
| **T_ACC** | 0.91 | 1.36 | 0.80 | 3.64 | 0.93 | 1.77 | 0.90 | 1.45 | 0.91 | 2.41 | 0.93 | 1.33 |
| **T_ACG** | 0.94 | 0.90 | 1.04 | 0.41 | 0.80 | 4.28 | 0.70 | 4.78 | 1.11 | 2.72 | 1.14 | 2.30 |
| **T_ACT** | 0.88 | 2.27 | 1.01 | 0.13 | 1.06 | 1.42 | 1.09 | 1.25 | 0.91 | 0.88 | 0.69 | 2.64 |
| **V_GTA** | 0.57 | 8.18 | 0.79 | 3.44 | 0.85 | 3.21 | 0.69 | 4.82 | 0.66 | 3.14 | 0.51 | 4.04 |
| **V_GTC** | 0.98 | 0.29 | 0.91 | 1.39 | 0.88 | 3.52 | 0.90 | 2.02 | 0.85 | 5.46 | 0.69 | 8.87 |
| **V_GTG** | 1.29 | 4.34 | 1.22 | 3.74 | 1.14 | 3.88 | 1.19 | 3.36 | 1.24 | 7.46 | 1.59 | 11.05 |
| **V_GTT** | 1.09 | 1.59 | 0.99 | 0.13 | 1.05 | 1.33 | 1.08 | 1.45 | 0.79 | 3.40 | 0.71 | 3.30 |
| **Y_TAC** | 1.32 | 2.16 | 1.05 | 0.34 | 1.39 | 4.04 | 1.00 | 0.05 | 1.89 | 3.80 | 1.59 | 1.64 |
| **Y_TAT** | 0.76 | 2.16 | 0.95 | 0.34 | 0.72 | 4.04 | 1.00 | 0.05 | 0.53 | 3.80 | 0.63 | 1.64 |
| **K_AAA** | 0.84 | 2.28 | 0.84 | 2.80 | 0.85 | 2.92 | 0.81 | 2.35 | 0.71 | 2.34 | 0.80 | 1.03 |
| **K_AAG** | 1.19 | 2.28 | 1.19 | 2.80 | 1.17 | 2.92 | 1.24 | 2.35 | 1.40 | 2.34 | 1.25 | 1.03 |

**Table S7 e:** Odds ratios for codon enrichment analysis, restricted to codons with the third base in a loop region. For each value, the corresponding significance is also reported.

| **Codon** | **AT** | **AT_sign** | **MT** | **MT_sign** | **OS(LGC)** | **OS(LGC)_sign** | **ZM(LGC)** | **ZM(LGC)_sign** | **OS(HGC)** | **OS(HGC)_sign** | **ZM(HGC)** | **ZM(HGC)_sign** |
| --- | --- | --- | --- | --- | --- | --- | --- | --- | --- | --- | --- | --- |
| **A_GCA** | 0.96 | 0.73 | 1.04 | 0.77 | 1.20 | 5.82 | 1.12 | 2.22 | 0.88 | 2.71 | 0.87 | 2.31 |
| **A_GCC** | 0.85 | 2.40 | 0.79 | 3.56 | 0.79 | 7.39 | 0.87 | 2.81 | 1.01 | 0.35 | 1.07 | 1.67 |
| **A_GCG** | 0.84 | 2.39 | 0.78 | 2.77 | 0.74 | 7.93 | 0.88 | 2.00 | 1.06 | 2.15 | 1.07 | 1.69 |
| **A_GCT** | 1.23 | 4.07 | 1.17 | 3.23 | 1.25 | 7.01 | 1.10 | 1.95 | 0.92 | 1.67 | 0.80 | 3.40 |
| **C_TGC** | 1.23 | 1.13 | 1.21 | 1.17 | 1.32 | 3.14 | 1.14 | 0.83 | 2.38 | 4.88 | 2.15 | 3.31 |
| **C_TGT** | 0.81 | 1.13 | 0.83 | 1.17 | 0.76 | 3.14 | 0.88 | 0.83 | 0.42 | 4.88 | 0.46 | 3.31 |
| **D_GAC** | 0.85 | 2.80 | 0.94 | 1.02 | 0.88 | 3.57 | 0.84 | 3.08 | 1.48 | 7.38 | 1.41 | 4.06 |
| **D_GAT** | 1.18 | 2.80 | 1.06 | 1.02 | 1.14 | 3.57 | 1.19 | 3.08 | 0.68 | 7.38 | 0.71 | 4.06 |
| **E_GAA** | 0.74 | 5.30 | 0.80 | 4.13 | 1.01 | 0.23 | 0.91 | 1.63 | 0.59 | 9.52 | 0.56 | 7.22 |
| **E_GAG** | 1.35 | 5.30 | 1.25 | 4.13 | 0.99 | 0.23 | 1.10 | 1.63 | 1.70 | 9.52 | 1.79 | 7.22 |
| **F_TTC** | 1.32 | 3.50 | 0.94 | 1.00 | 1.13 | 2.25 | 1.09 | 0.95 | 1.43 | 3.31 | 1.65 | 3.51 |
| **F_TTT** | 0.76 | 3.50 | 1.07 | 1.00 | 0.88 | 2.25 | 0.92 | 0.95 | 0.70 | 3.31 | 0.60 | 3.51 |
| **G_GGA** | 1.49 | 5.12 | 1.53 | 6.26 | 1.47 | 8.79 | 1.29 | 3.67 | 0.94 | 0.91 | 1.00 | 0.00 |
| **G_GGC** | 0.77 | 2.65 | 0.82 | 1.96 | 0.73 | 7.12 | 0.80 | 3.07 | 0.98 | 0.37 | 0.95 | 0.91 |
| **G_GGG** | 1.22 | 1.79 | 1.19 | 1.75 | 1.05 | 0.88 | 1.24 | 2.53 | 1.22 | 4.29 | 1.21 | 2.80 |
| **G_GGT** | 0.70 | 4.79 | 0.68 | 6.17 | 0.87 | 3.17 | 0.80 | 3.13 | 0.70 | 5.11 | 0.72 | 2.98 |
| **H_CAC** | 1.00 | 0.00 | 0.93 | 0.91 | 1.07 | 1.24 | 0.83 | 2.19 | 1.65 | 6.54 | 1.81 | 4.81 |
| **H_CAT** | 1.00 | 0.00 | 1.08 | 0.91 | 0.94 | 1.24 | 1.21 | 2.19 | 0.60 | 6.54 | 0.55 | 4.81 |
| **I_ATA** | 0.77 | 4.88 | 0.95 | 1.01 | 0.93 | 2.00 | 1.02 | 0.23 | 0.89 | 1.45 | 0.95 | 0.39 |
| **I_ATC** | 1.42 | 6.23 | 0.94 | 1.07 | 1.09 | 2.22 | 1.08 | 1.24 | 1.29 | 4.36 | 1.28 | 2.73 |
| **I_ATT** | 0.94 | 1.26 | 1.08 | 1.80 | 0.99 | 0.13 | 0.92 | 1.40 | 0.74 | 4.07 | 0.68 | 3.22 |
| **L_CTA** | 0.99 | 0.08 | 1.01 | 0.13 | 1.13 | 2.43 | 1.05 | 0.64 | 0.76 | 3.00 | 0.87 | 1.09 |
| **L_CTC** | 1.15 | 1.91 | 0.92 | 1.36 | 0.90 | 2.45 | 1.12 | 1.82 | 1.21 | 4.91 | 1.34 | 5.82 |
| **L_CTG** | 1.06 | 0.61 | 1.04 | 0.35 | 1.11 | 2.29 | 1.08 | 1.08 | 1.22 | 4.59 | 1.06 | 1.12 |
| **L_CTT** | 1.23 | 3.37 | 1.28 | 4.90 | 1.15 | 3.64 | 1.19 | 2.83 | 0.93 | 0.96 | 0.72 | 3.33 |
| **L_TTA** | 0.75 | 4.66 | 0.73 | 5.85 | 0.87 | 2.93 | 0.69 | 5.20 | 0.43 | 5.90 | 0.31 | 5.94 |
| **L_TTG** | 0.92 | 1.31 | 1.06 | 0.97 | 0.86 | 3.59 | 0.85 | 2.46 | 0.41 | 12.98 | 0.44 | 8.41 |
| **N_AAC** | 1.27 | 4.43 | 0.97 | 0.64 | 1.11 | 2.85 | 1.05 | 0.75 | 1.62 | 6.98 | 1.69 | 4.45 |
| **N_AAT** | 0.79 | 4.43 | 1.03 | 0.64 | 0.90 | 2.85 | 0.95 | 0.75 | 0.62 | 6.98 | 0.59 | 4.45 |
| **P_CCA** | 1.09 | 1.18 | 0.96 | 0.54 | 1.07 | 1.56 | 1.09 | 1.22 | 0.90 | 1.63 | 0.95 | 0.50 |
| **P_CCC** | 0.74 | 2.97 | 0.75 | 3.36 | 0.84 | 3.42 | 0.86 | 1.87 | 1.03 | 0.57 | 0.99 | 0.10 |
| **P_CCG** | 0.90 | 1.05 | 1.08 | 0.58 | 0.78 | 4.52 | 0.88 | 1.38 | 1.12 | 2.50 | 1.18 | 2.73 |
| **P_CCT** | 1.12 | 1.50 | 1.17 | 2.53 | 1.23 | 4.67 | 1.10 | 1.38 | 0.82 | 2.96 | 0.74 | 3.42 |
| **Q_CAA** | 0.98 | 0.21 | 0.71 | 5.20 | 0.85 | 3.79 | 0.90 | 1.41 | 0.58 | 7.94 | 0.56 | 5.48 |
| **Q_CAG** | 1.02 | 0.21 | 1.42 | 5.20 | 1.17 | 3.79 | 1.11 | 1.41 | 1.73 | 7.94 | 1.77 | 5.48 |
| **R_AGA** | 0.90 | 1.56 | 0.93 | 1.04 | 0.95 | 1.26 | 0.87 | 2.16 | 0.60 | 6.44 | 0.76 | 2.60 |
| **R_AGG** | 0.89 | 1.33 | 1.02 | 0.22 | 0.99 | 0.31 | 0.92 | 1.13 | 0.62 | 9.81 | 0.60 | 7.71 |
| **R_CGA** | 1.24 | 2.05 | 0.94 | 0.62 | 1.01 | 0.15 | 1.00 | 0.04 | 0.77 | 2.68 | 0.75 | 2.34 |
| **R_CGC** | 0.97 | 0.14 | 1.18 | 1.33 | 1.06 | 0.97 | 1.13 | 1.45 | 1.47 | 8.34 | 1.50 | 6.52 |
| **R_CGG** | 1.16 | 1.01 | 1.02 | 0.11 | 1.16 | 2.36 | 1.32 | 2.62 | 1.41 | 6.54 | 1.35 | 4.39 |
| **R_CGT** | 1.09 | 0.91 | 1.05 | 0.55 | 0.92 | 1.37 | 1.04 | 0.38 | 0.87 | 1.31 | 0.70 | 2.71 |
| **S_AGC** | 0.76 | 4.35 | 0.92 | 1.20 | 0.75 | 7.98 | 0.75 | 4.94 | 0.81 | 5.52 | 0.69 | 7.02 |
| **S_AGT** | 0.45 | 16.44 | 0.74 | 6.18 | 0.68 | 10.46 | 0.56 | 9.48 | 0.58 | 5.96 | 0.37 | 7.52 |
| **S_TCA** | 1.42 | 6.68 | 1.14 | 3.16 | 1.35 | 9.53 | 1.59 | 8.67 | 0.98 | 0.30 | 1.23 | 2.08 |
| **S_TCC** | 1.11 | 1.57 | 0.86 | 2.70 | 1.06 | 1.64 | 1.09 | 1.49 | 1.19 | 5.15 | 1.31 | 5.55 |
| **S_TCG** | 1.08 | 1.05 | 0.94 | 0.82 | 0.90 | 2.28 | 0.92 | 1.07 | 1.14 | 3.35 | 1.21 | 3.24 |
| **S_TCT** | 1.49 | 7.90 | 1.23 | 4.96 | 1.21 | 5.66 | 1.14 | 2.37 | 0.88 | 1.76 | 0.92 | 0.79 |
| **T_ACA** | 1.27 | 4.65 | 1.13 | 2.71 | 1.24 | 6.20 | 1.49 | 6.89 | 0.91 | 1.45 | 0.85 | 1.78 |
| **T_ACC** | 0.93 | 1.15 | 0.76 | 4.99 | 0.81 | 5.05 | 0.75 | 4.57 | 1.00 | 0.06 | 1.00 | 0.05 |
| **T_ACG** | 0.95 | 0.71 | 0.73 | 3.71 | 0.83 | 3.33 | 0.84 | 2.08 | 1.09 | 1.89 | 1.19 | 2.89 |
| **T_ACT** | 0.85 | 3.17 | 1.17 | 3.33 | 1.01 | 0.18 | 0.92 | 1.43 | 0.85 | 1.88 | 0.71 | 3.06 |
| **V_GTA** | 0.70 | 6.27 | 0.71 | 6.06 | 0.80 | 5.31 | 0.82 | 3.02 | 0.59 | 5.69 | 0.58 | 4.72 |
| **V_GTC** | 1.03 | 0.36 | 0.99 | 0.18 | 1.02 | 0.59 | 0.86 | 2.63 | 0.91 | 2.47 | 0.74 | 5.91 |
| **V_GTG** | 1.17 | 2.48 | 1.20 | 3.01 | 1.08 | 1.93 | 1.23 | 3.51 | 1.24 | 5.90 | 1.64 | 9.53 |
| **V_GTT** | 1.14 | 2.68 | 1.12 | 2.50 | 1.07 | 1.97 | 1.08 | 1.52 | 0.86 | 2.28 | 0.75 | 3.03 |
| **Y_TAC** | 1.46 | 3.77 | 1.02 | 0.23 | 1.15 | 2.21 | 1.21 | 1.85 | 1.72 | 4.19 | 1.35 | 1.67 |
| **Y_TAT** | 0.69 | 3.77 | 0.98 | 0.23 | 0.87 | 2.21 | 0.82 | 1.85 | 0.58 | 4.19 | 0.74 | 1.67 |
| **K_AAA** | 0.72 | 6.16 | 0.79 | 5.27 | 0.81 | 6.27 | 0.70 | 6.13 | 0.81 | 3.10 | 0.77 | 2.45 |
| **K_AAG** | 1.40 | 6.16 | 1.27 | 5.27 | 1.24 | 6.27 | 1.42 | 6.13 | 1.23 | 3.10 | 1.29 | 2.45 |

**Table S7 f:** Odds ratios for codon enrichment analysis relative to the Mantel Heisenzl test between conserved sites in stems and conserved sites in loops. For each value, the corresponding significance is also reported.

| **Codon** | **AT** | **AT_sign** | **MT** | **MT_sign** | **OS(LGC)** | **OS(LGC)_sign** | **ZM(LGC)** | **ZM(LGC)_sign** | **OS(HGC)** | **OS(HGC)_sign** | **ZM(HGC)** | **ZM(HGC)_sign** |
| --- | --- | --- | --- | --- | --- | --- | --- | --- | --- | --- | --- | --- |
| **A_GCA** | 0.67 | 21.64 | 0.75 | 9.42 | 0.65 | 21.01 | 0.65 | 12.93 | 0.34 | 27.12 | 0.35 | 19.00 |
| **A_GCC** | 1.17 | 6.95 | 1.27 | 6.04 | 1.12 | 5.41 | 1.04 | 1.26 | 0.89 | 6.72 | 0.86 | 5.79 |
| **A_GCG** | 1.36 | 12.84 | 1.32 | 5.12 | 1.59 | 18.65 | 1.66 | 12.80 | 1.53 | 23.38 | 1.58 | 17.53 |
| **A_GCT** | 1.09 | 5.29 | 1.05 | 1.86 | 1.00 | 0.17 | 1.02 | 0.73 | 0.69 | 9.76 | 0.74 | 5.91 |
| **C_TGC** | 1.26 | 6.57 | 1.28 | 4.04 | 1.26 | 5.45 | 1.37 | 4.45 | 1.41 | 3.61 | 1.68 | 3.73 |
| **C_TGT** | 0.79 | 6.57 | 0.78 | 4.04 | 0.80 | 5.45 | 0.73 | 4.45 | 0.71 | 3.61 | 0.59 | 3.73 |
| **D_GAC** | 1.07 | 3.26 | 1.05 | 1.43 | 1.04 | 1.73 | 1.10 | 2.63 | 1.18 | 4.16 | 1.16 | 2.20 |
| **D_GAT** | 0.94 | 3.26 | 0.95 | 1.43 | 0.96 | 1.73 | 0.91 | 2.63 | 0.85 | 4.16 | 0.86 | 2.20 |
| **E_GAA** | 0.38 | 57.49 | 0.40 | 31.32 | 0.31 | 54.72 | 0.29 | 35.03 | 0.25 | 29.07 | 0.20 | 21.18 |
| **E_GAG** | 2.66 | 57.49 | 2.53 | 31.32 | 3.20 | 54.72 | 3.42 | 35.03 | 4.02 | 29.07 | 5.09 | 21.18 |
| **F_TTC** | 1.26 | 10.73 | 1.27 | 6.73 | 1.37 | 11.73 | 1.29 | 6.05 | 1.32 | 4.33 | 1.32 | 3.05 |
| **F_TTT** | 0.80 | 10.73 | 0.79 | 6.73 | 0.73 | 11.73 | 0.77 | 6.05 | 0.76 | 4.33 | 0.76 | 3.05 |
| **G_GGA** | 0.74 | 17.61 | 0.69 | 12.96 | 0.57 | 25.78 | 0.55 | 16.91 | 0.44 | 21.92 | 0.40 | 17.10 |
| **G_GGC** | 1.19 | 7.25 | 1.26 | 5.70 | 1.32 | 12.34 | 1.42 | 9.75 | 1.27 | 11.19 | 1.28 | 8.04 |
| **G_GGG** | 1.30 | 11.50 | 1.33 | 7.46 | 1.41 | 14.35 | 1.31 | 6.98 | 1.07 | 2.89 | 1.13 | 3.56 |
| **G_GGT** | 1.07 | 3.86 | 1.09 | 3.11 | 1.00 | 0.14 | 1.02 | 0.45 | 0.84 | 4.18 | 0.77 | 4.22 |
| **H_CAC** | 0.92 | 2.62 | 0.89 | 1.99 | 0.84 | 4.67 | 1.07 | 1.01 | 1.03 | 0.43 | 1.11 | 0.90 |
| **H_CAT** | 1.09 | 2.62 | 1.12 | 1.99 | 1.19 | 4.67 | 0.94 | 1.01 | 0.97 | 0.43 | 0.90 | 0.90 |
| **I_ATA** | 0.52 | 27.75 | 0.54 | 15.18 | 0.48 | 24.19 | 0.50 | 14.57 | 0.27 | 16.88 | 0.26 | 12.05 |
| **I_ATC** | 1.61 | 23.34 | 1.49 | 9.93 | 1.62 | 18.37 | 1.73 | 12.77 | 2.09 | 14.15 | 2.58 | 11.70 |
| **I_ATT** | 1.03 | 1.36 | 1.17 | 4.66 | 1.10 | 3.58 | 1.02 | 0.55 | 0.86 | 2.29 | 0.72 | 3.12 |
| **L_CTA** | 0.50 | 29.83 | 0.52 | 16.22 | 0.40 | 31.72 | 0.44 | 17.80 | 0.24 | 20.32 | 0.28 | 14.41 |
| **L_CTC** | 1.15 | 7.63 | 1.03 | 0.92 | 1.12 | 5.70 | 1.06 | 1.85 | 0.78 | 13.55 | 0.66 | 15.56 |
| **L_CTG** | 1.52 | 18.46 | 1.43 | 8.77 | 1.49 | 20.31 | 1.64 | 15.72 | 1.54 | 22.42 | 1.83 | 22.48 |
| **L_CTT** | 0.97 | 1.68 | 0.96 | 1.61 | 0.92 | 4.29 | 0.83 | 6.00 | 0.71 | 8.06 | 0.72 | 5.23 |
| **L_TTA** | 0.50 | 33.64 | 0.51 | 19.34 | 0.42 | 27.39 | 0.48 | 14.81 | 0.25 | 10.78 | 0.19 | 7.20 |
| **L_TTG** | 1.67 | 31.24 | 1.74 | 21.27 | 1.52 | 20.24 | 1.46 | 11.40 | 1.37 | 7.43 | 1.24 | 2.98 |
| **N_AAC** | 1.12 | 5.08 | 1.15 | 3.42 | 1.06 | 1.88 | 1.12 | 2.27 | 1.08 | 1.15 | 1.36 | 2.79 |
| **N_AAT** | 0.90 | 5.08 | 0.87 | 3.42 | 0.95 | 1.88 | 0.90 | 2.27 | 0.93 | 1.15 | 0.73 | 2.79 |
| **P_CCA** | 0.72 | 15.73 | 0.82 | 5.86 | 0.69 | 15.78 | 0.71 | 8.70 | 0.34 | 25.61 | 0.36 | 16.13 |
| **P_CCC** | 0.91 | 2.94 | 0.94 | 1.31 | 0.87 | 4.64 | 0.91 | 2.12 | 0.56 | 22.23 | 0.58 | 14.65 |
| **P_CCG** | 1.95 | 26.38 | 1.82 | 11.25 | 1.84 | 20.87 | 1.74 | 11.87 | 2.62 | 40.31 | 2.48 | 25.95 |
| **P_CCT** | 0.93 | 3.50 | 0.99 | 0.25 | 1.06 | 2.53 | 1.02 | 0.58 | 0.68 | 9.15 | 0.70 | 5.79 |
| **Q_CAA** | 0.35 | 43.50 | 0.37 | 23.46 | 0.31 | 39.37 | 0.29 | 24.71 | 0.19 | 21.91 | 0.16 | 14.82 |
| **Q_CAG** | 2.86 | 43.50 | 2.69 | 23.46 | 3.24 | 39.37 | 3.40 | 24.71 | 5.29 | 21.91 | 6.37 | 14.82 |
| **R_AGA** | 0.67 | 20.64 | 0.66 | 12.02 | 0.52 | 25.00 | 0.49 | 16.57 | 0.35 | 18.04 | 0.32 | 13.06 |
| **R_AGG** | 1.55 | 18.99 | 1.68 | 13.70 | 1.57 | 18.63 | 1.56 | 11.22 | 1.19 | 6.19 | 1.20 | 4.21 |
| **R_CGA** | 0.76 | 9.82 | 0.76 | 5.35 | 0.58 | 14.46 | 0.57 | 8.89 | 0.39 | 14.67 | 0.37 | 10.30 |
| **R_CGC** | 1.16 | 4.04 | 1.06 | 1.01 | 1.17 | 5.50 | 1.23 | 4.53 | 0.97 | 1.09 | 0.95 | 1.36 |
| **R_CGG** | 1.73 | 17.18 | 1.65 | 7.99 | 1.54 | 14.14 | 1.63 | 9.82 | 1.31 | 10.60 | 1.38 | 8.87 |
| **R_CGT** | 0.96 | 1.45 | 0.91 | 2.35 | 0.89 | 3.33 | 0.84 | 3.20 | 0.76 | 4.72 | 0.69 | 4.18 |
| **S_AGC** | 1.49 | 18.80 | 1.60 | 10.89 | 1.49 | 15.88 | 1.46 | 9.28 | 1.35 | 11.51 | 1.34 | 7.78 |
| **S_AGT** | 1.12 | 5.72 | 1.15 | 4.12 | 1.05 | 1.84 | 1.12 | 2.40 | 0.90 | 1.34 | 0.88 | 0.93 |
| **S_TCA** | 0.58 | 30.93 | 0.66 | 14.03 | 0.59 | 23.88 | 0.56 | 15.95 | 0.33 | 19.81 | 0.34 | 12.94 |
| **S_TCC** | 1.11 | 4.73 | 1.16 | 3.86 | 1.06 | 2.27 | 1.07 | 1.84 | 0.71 | 14.05 | 0.72 | 9.70 |
| **S_TCG** | 1.27 | 10.73 | 1.17 | 2.97 | 1.33 | 9.53 | 1.27 | 4.92 | 1.53 | 16.75 | 1.55 | 11.63 |
| **S_TCT** | 0.98 | 1.20 | 0.96 | 1.44 | 0.99 | 0.41 | 1.02 | 0.46 | 0.63 | 8.23 | 0.62 | 5.95 |
| **T_ACA** | 0.57 | 26.59 | 0.65 | 11.65 | 0.58 | 19.58 | 0.56 | 12.33 | 0.34 | 16.65 | 0.36 | 10.84 |
| **T_ACC** | 1.26 | 9.61 | 1.35 | 6.75 | 1.27 | 7.95 | 1.10 | 1.89 | 0.68 | 12.95 | 0.66 | 9.71 |
| **T_ACG** | 1.55 | 16.18 | 1.58 | 6.84 | 1.64 | 12.87 | 1.62 | 7.83 | 2.03 | 23.06 | 2.09 | 16.82 |
| **T_ACT** | 1.12 | 5.54 | 1.08 | 2.21 | 1.10 | 3.41 | 1.26 | 4.94 | 0.77 | 3.71 | 0.64 | 4.37 |
| **V_GTA** | 0.54 | 25.96 | 0.61 | 12.07 | 0.50 | 22.01 | 0.47 | 14.67 | 0.34 | 13.06 | 0.31 | 10.21 |
| **V_GTC** | 1.23 | 9.87 | 1.08 | 1.92 | 1.16 | 6.06 | 1.20 | 4.58 | 0.82 | 8.75 | 0.76 | 8.20 |
| **V_GTG** | 1.55 | 22.88 | 1.72 | 16.24 | 1.66 | 22.81 | 1.65 | 13.93 | 1.49 | 18.14 | 1.58 | 14.17 |
| **V_GTT** | 0.85 | 9.46 | 0.83 | 6.61 | 0.76 | 12.47 | 0.76 | 7.71 | 0.53 | 13.14 | 0.58 | 7.36 |
| **Y_TAC** | 1.00 | 0.10 | 0.99 | 0.23 | 1.02 | 0.51 | 0.97 | 0.50 | 1.17 | 2.06 | 1.40 | 2.85 |
| **Y_TAT** | 1.00 | 0.10 | 1.01 | 0.23 | 0.98 | 0.51 | 1.03 | 0.50 | 0.86 | 2.06 | 0.71 | 2.85 |
| **K_AAA** | 0.32 | 61.63 | 0.33 | 35.83 | 0.29 | 50.40 | 0.28 | 31.15 | 0.19 | 24.15 | 0.23 | 15.19 |
| **K_AAG** | 3.10 | 61.63 | 3.03 | 35.83 | 3.50 | 50.40 | 3.61 | 31.15 | 5.26 | 24.15 | 4.43 | 15.19 |
