# Supplementary material for: The Evolutionary Basis of Translational Accuracy in Plants
Source: G3 (Bethesda). 2017 May 22;7(7):2363–73. doi: 10.1534/g3.117.040626 (PMC5499143; doi:10.1534/g3.117.040626)
Supplement: Supplementary file 8 [file 2363TableS8.docx]

**Table S8:** Translational accuracy test results. Odds ratio were obtained by performing an optimality-tied Akashi test (see Methods).

| **Species** | **Odds ratio** | | | | |
| --- | --- | --- | --- | --- | --- |
|  |  |  |  |  |  |
|  | *All* | *Domains* | *Non-domains* | *Stems* | *Loops* |
| **AT** | 1.1 | 1.12 | 1.07 | 1.09 | 1.13 |
| **MT** | 1.02 | 1.06 | 1.01ns | 0.99ns | 1.03 |
| **OS_HGC** | 1.1 | 1.01ns | 1.08 | 1.04 | 1.14 |
| **OS_LGC** | 1.08 | 1.03 | 1.04 | 1.11 | 1.08 |
| **ZM_HGC** | 0.75 | 0.81 | 0.75 | 0.78 | 0.74 |
| **ZM_LGC** | 1.1 | 1.02 | 1.07 | 1.13 | 1.09 |
