# Supplementary material for: The Evolutionary Basis of Translational Accuracy in Plants
Source: G3 (Bethesda). 2017 May 22;7(7):2363–73. doi: 10.1534/g3.117.040626 (PMC5499143; doi:10.1534/g3.117.040626)
Supplement: Supplementary file 9 [file 2363TableS9.docx]

| **Species** | **Spearman p** |
| --- | --- |
| AT | 0.2828* |
| MT | 0.3764* |
| OS(HGC) | -0.0573 |
| OS(LGC) | 0.3211* |
| ZM(HGC) | 0.289* |
| ZM(LGC) | -0.07 |

**Table S9:** Correlation of differeneces observed between the odds ratios in protein domain and non-domain regions for each codon and the corresponding odds ratio values following the computation of optimal codons (* p<0.05)
