# Supplementary figures and images for: The Evolutionary Basis of Translational Accuracy in Plants
Source: G3 (Bethesda). 2017 May 22;7(7):2363–73. doi: 10.1534/g3.117.040626 (PMC5499143; doi:10.1534/g3.117.040626)

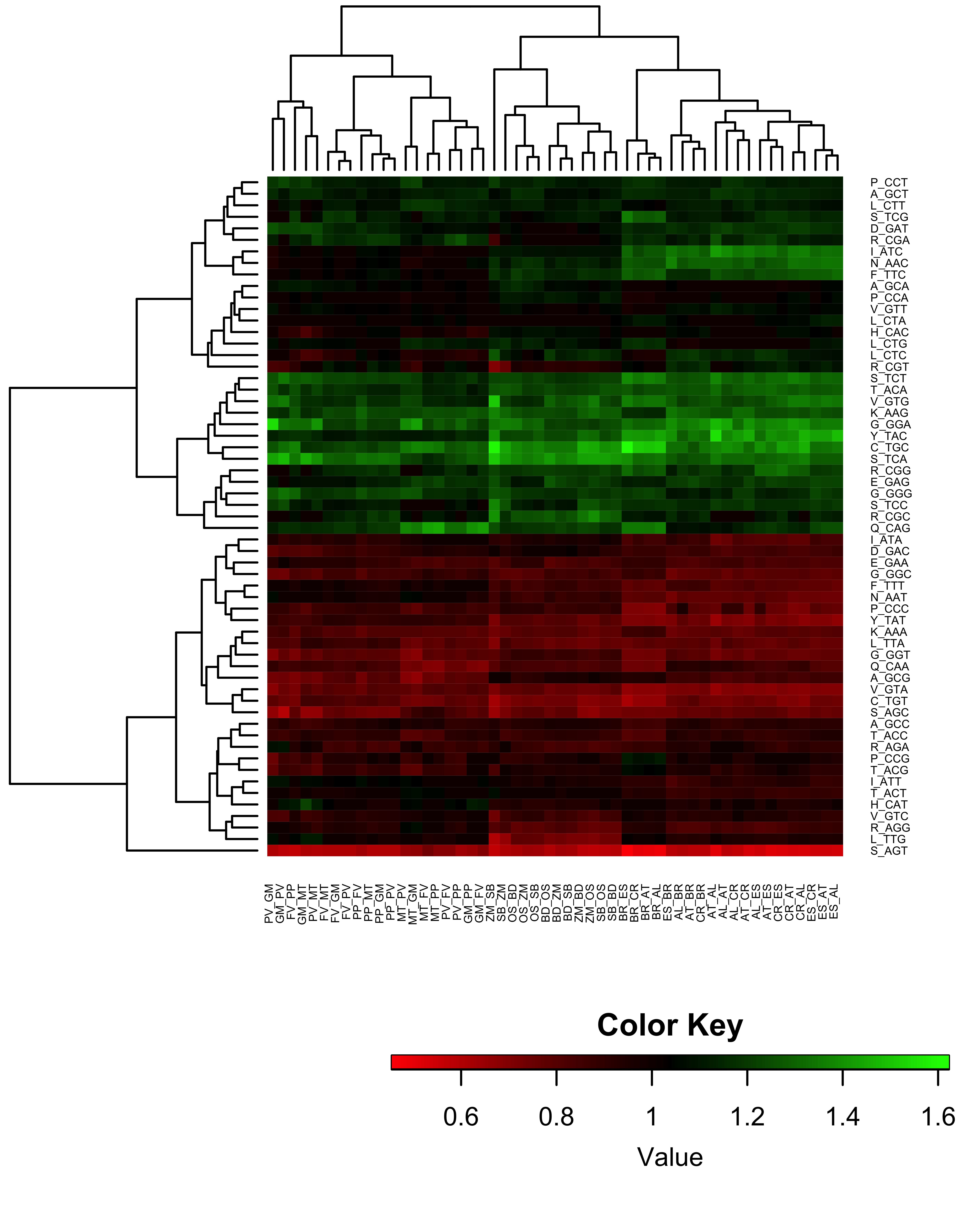

Supplement: Supplementary file 10 [file 2363FigureS1.jpg]

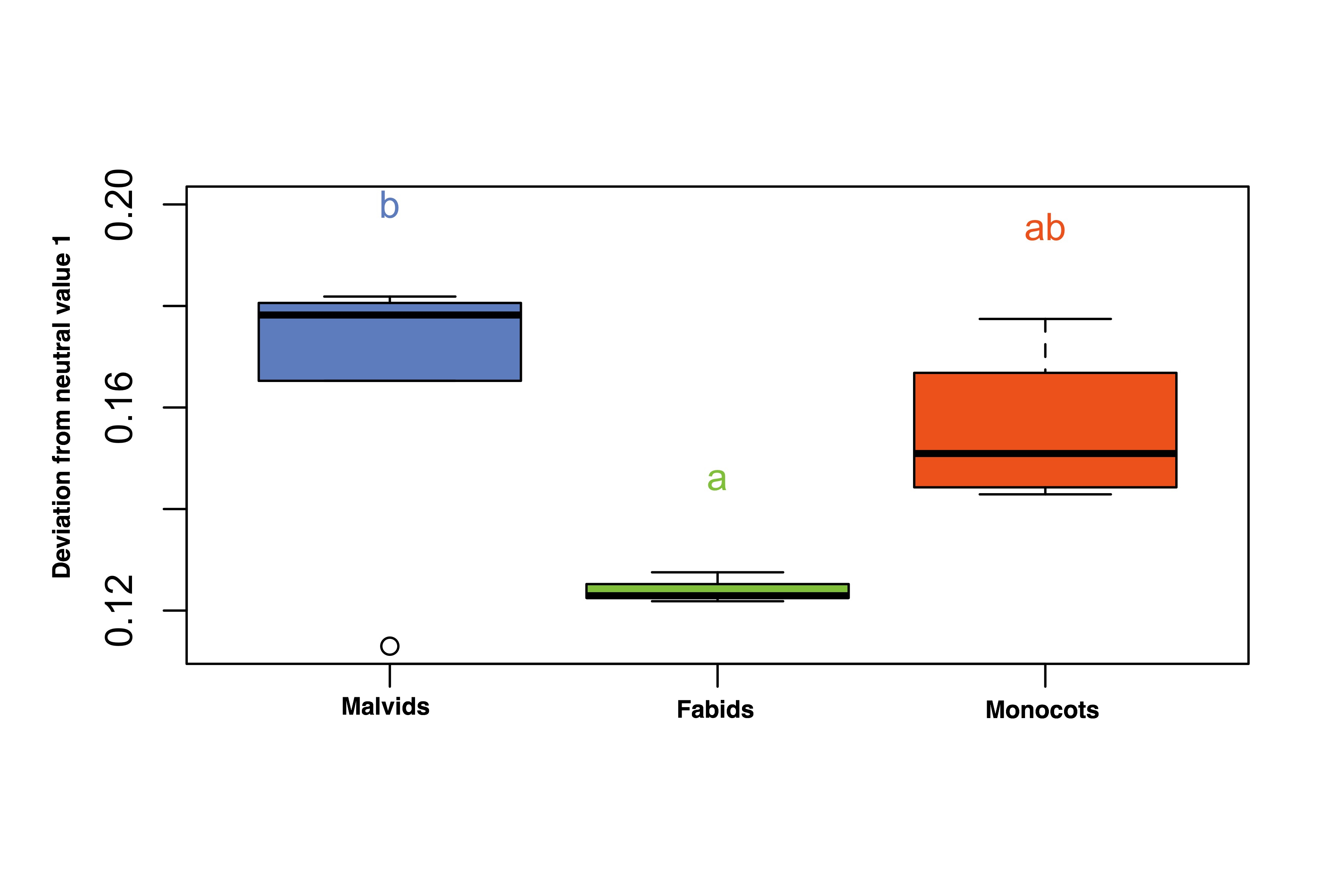

Supplement: Supplementary file 11 [file 2363FigureS2.jpg]

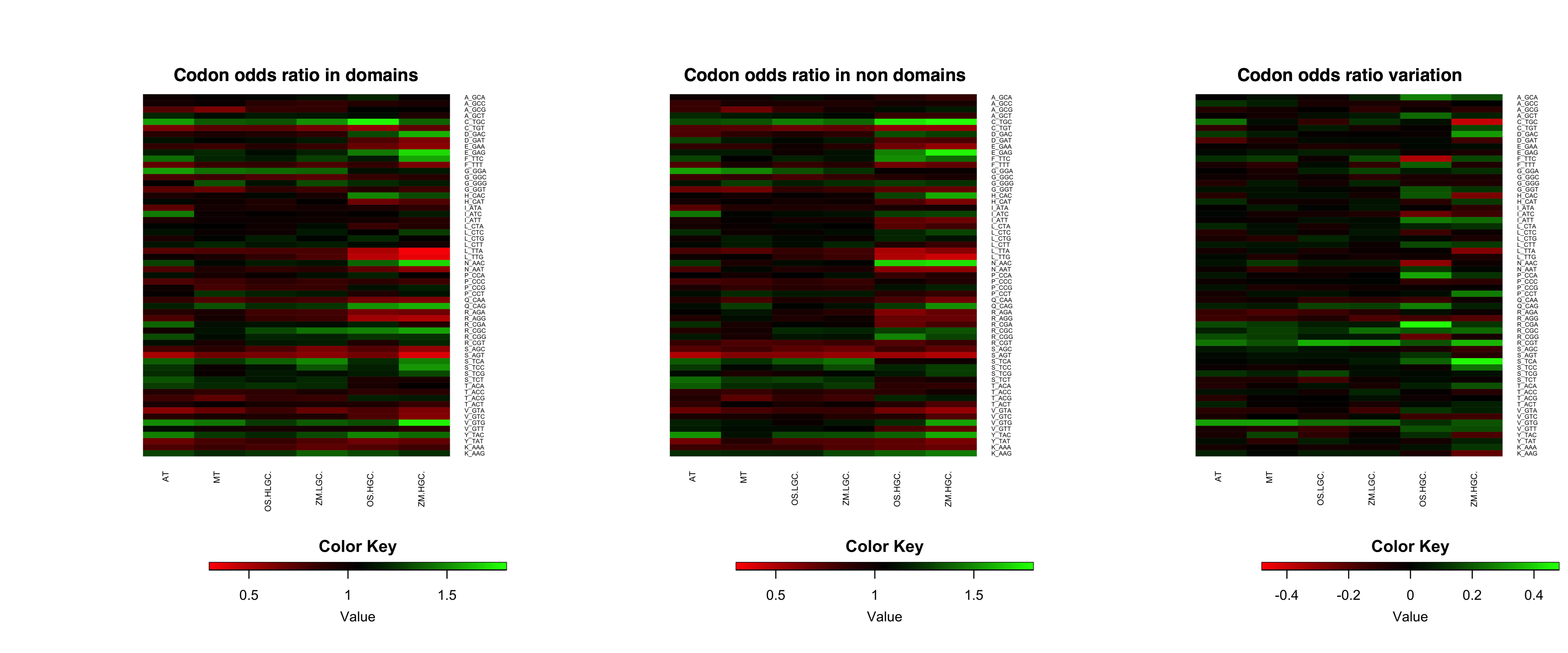

Supplement: Supplementary file 12 [file 2363FigureS3.jpg]

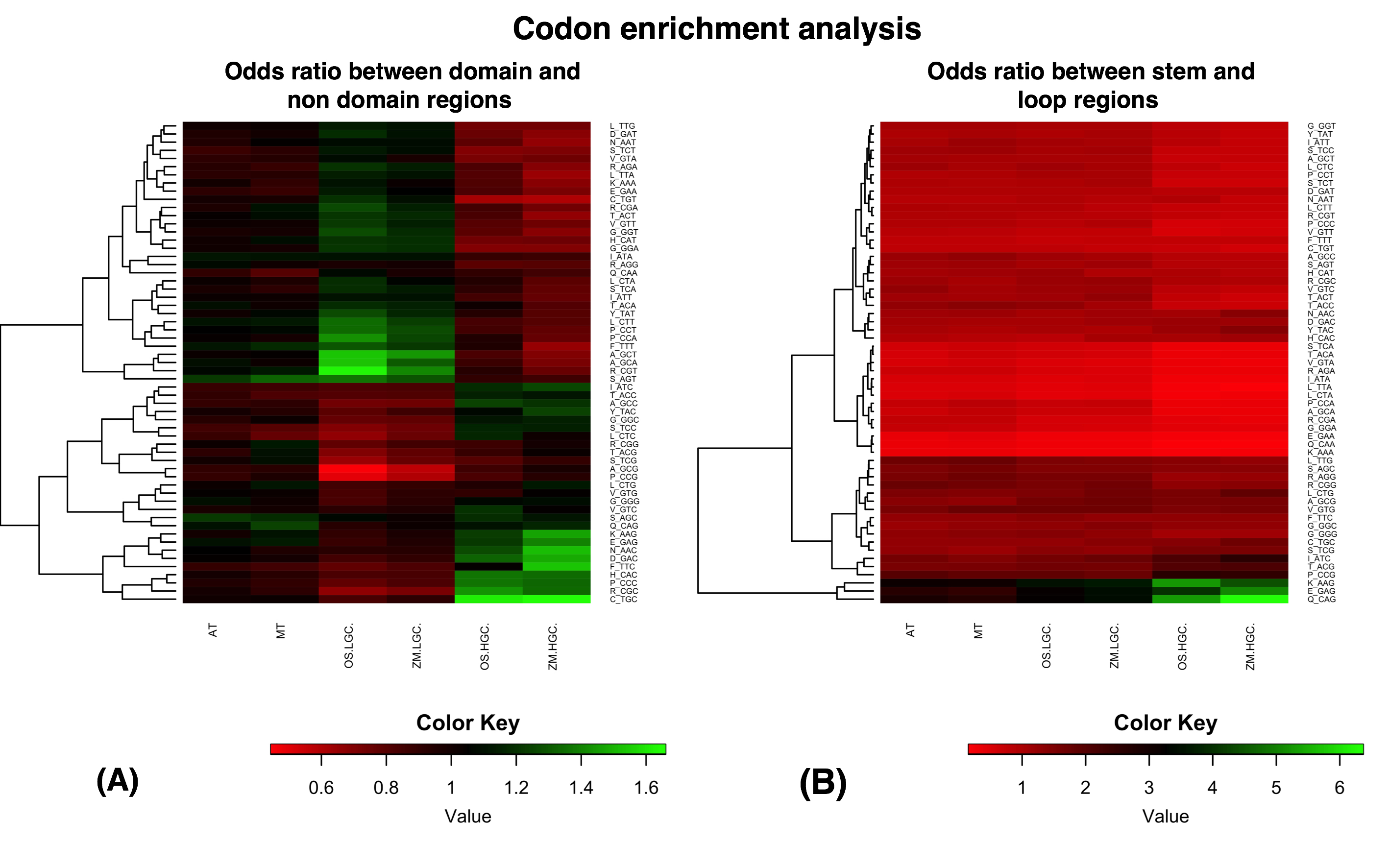

Supplement: Supplementary file 13 [file 2363FigureS4.jpg]

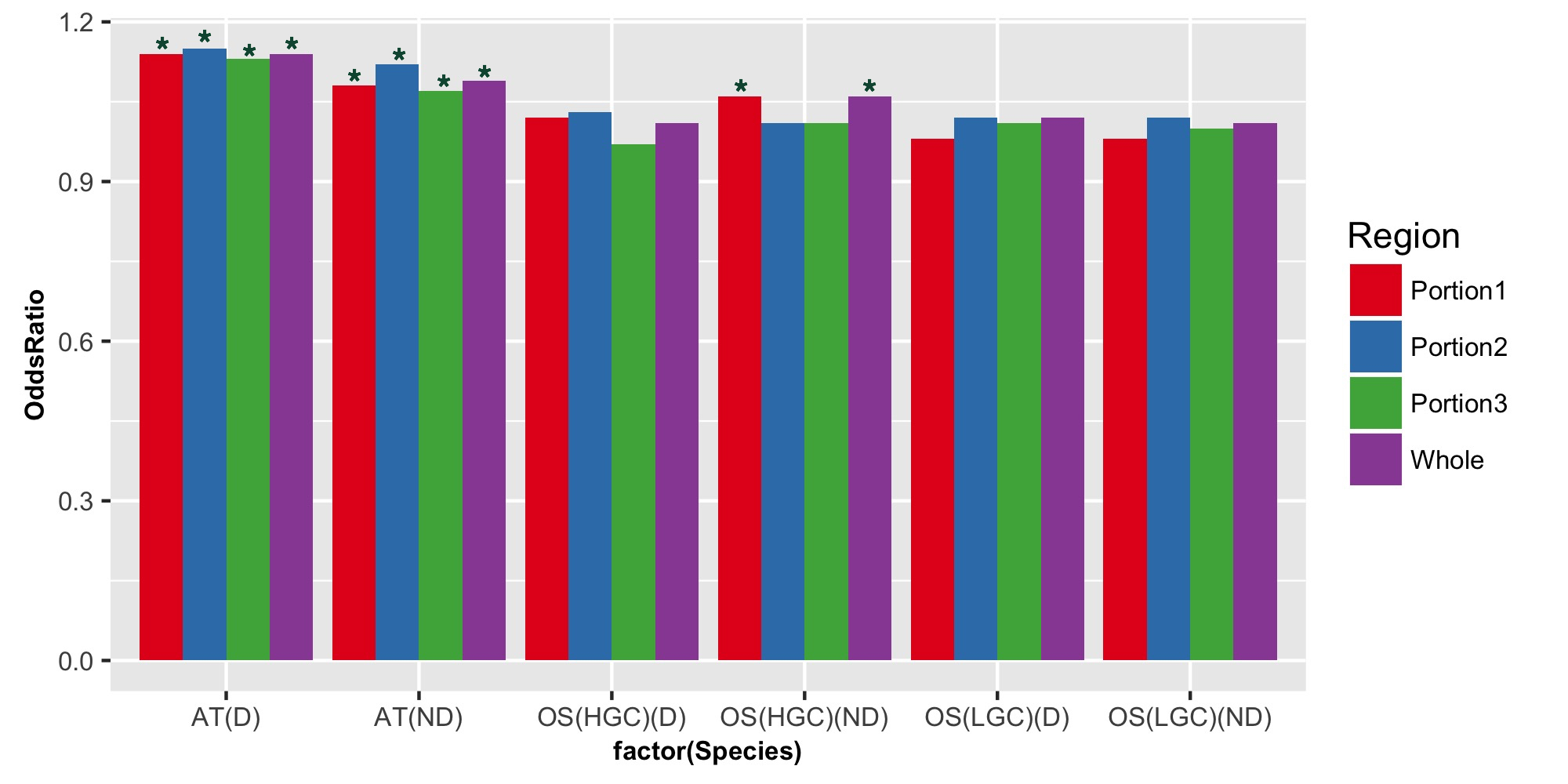

Supplement: Supplementary file 14 [file 2363FigureS5.jpg]

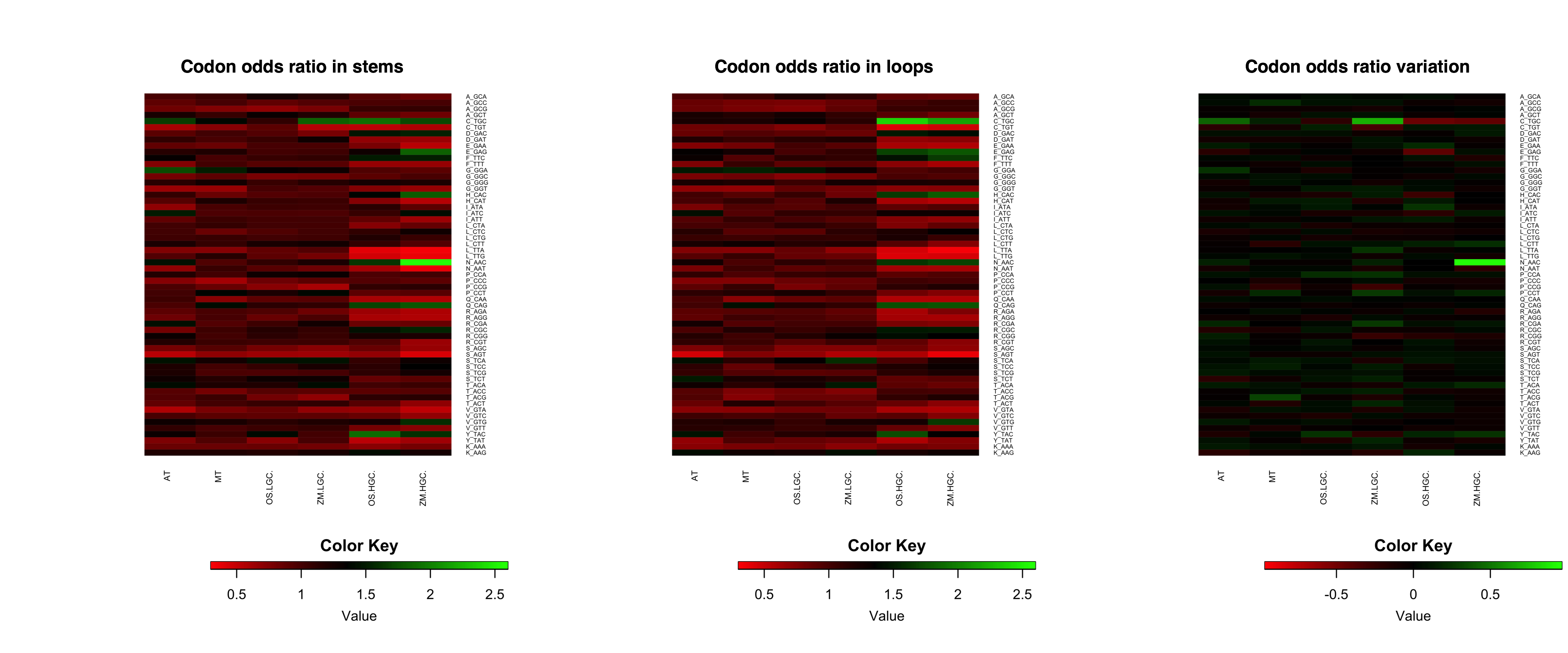

Supplement: Supplementary file 15 [file 2363FigureS6.jpg]
