# Supplementary material for: The Evolutionary Basis of Translational Accuracy in Plants
Source: G3 (Bethesda). 2017 May 22;7(7):2363–73. doi: 10.1534/g3.117.040626 (PMC5499143; doi:10.1534/g3.117.040626)
Supplement: Supplementary file 16 [file 2363FileS1.docx]

**Detailed description of the supplemental files**

**Supplemental file 2**

**Name:** Supplemental_File_2.txt

**Format:** text

**Description:** Orthologues gene couples identified for all the analysed brassicaceae, fabidae and monocots as described in the Materials and methods section

Supplemental file 3

Name: Supplemental_File_3.txt

Format: text

Description: 1:1 orthologues genes within each clade that have been used to extrapolate the species triplets for the “phylogeny-based dataset” (see Material and methods)

Supplemental file 4

Name: Supplemental_File_4.txt

Format: text

Description: Coordinates of proteins domains for all the analysed species

Supplemental file 5

Name: Supplemental_File_5.txt

Format: text

Description: Pseudo-alignments file created by considering the positions of stems and loops within each mRNA for all the analysed species. Briefly, transcripts’ positions involved in stems and loops have been identified by RNAfold with default parameters. For each coding sequence, a fasta formatted alignment has been generated featuring the original coded protein aligned to a copy featuring the same amino acid when the third base of the corresponding codon was involved in a stem structure and a letter “J” otherwise. Such a shortcut allowed the analysis of the enrichment in optimal codons in stems as compared to loops with the software Seforta (in this setting non conserved residues are computed at positions featuring the letter “J” in the pseudo-alignment).
